# Evolutionarily conserved sites in yeast tropomyosin function in cell polarity, transport and contractile ring formation

**DOI:** 10.1242/bio.012609

**Published:** 2015-07-17

**Authors:** Susanne Cranz-Mileva, Brittany MacTaggart, Jacquelyn Russell, Sarah E. Hitchcock-DeGregori

**Affiliations:** Department of Pathology and Laboratory Medicine, Robert Wood Johnson Medical School, Rutgers University, Piscataway, NJ 08854, USA

**Keywords:** Actin cytoskeleton, *Schizosaccharomyces pombe*, Fission yeast, *cdc8*, Actin binding protein, LifeAct, Myosin, Fimbrin

## Abstract

Tropomyosin is a coiled-coil protein that binds and regulates actin filaments. The tropomyosin gene in *Schizosaccharomyces pombe*, *cdc8*, is required for formation of actin cables, contractile rings, and polar localization of actin patches. The roles of conserved residues were investigated in gene replacement mutants. The work validates an evolution-based approach to identify tropomyosin functions in living cells and sites of potential interactions with other proteins. A *cdc8* mutant with near-normal actin affinity affects patch polarization and vacuole fusion, possibly by affecting Myo52p, a class V myosin, function. The presence of labile residual cell attachments suggests a delay in completion of cell division and redistribution of cell patches following cytokinesis. Another mutant with a mild phenotype is synthetic negative with GFP-fimbrin, inferring involvement of the mutated tropomyosin sites in interaction between the two proteins. Proteins that assemble in the contractile ring region before actin do so in a mutant *cdc8* strain that cannot assemble condensed actin rings, yet some cells can divide. Of general significance, LifeAct-GFP negatively affects the actin cytoskeleton, indicating caution in its use as a biomarker for actin filaments.

## INTRODUCTION

Actin-based cellular mechanisms in cytokinesis, intracellular transport, and establishment of cellular polarity in eukaryotes are universal. The deciphering of signaling pathways that regulate the cytoskeleton has been a focus whereas regulation at the terminal machinery has received less attention. Tropomyosin is a core actin regulatory protein, known for its role in regulating muscle contraction (Lehman and Craig, 2008), and is found in most eukaryotes. It is a two-chained α-helical coiled-coil protein that binds end-to-end along the length of both sides of the actin filament. In this position, tropomyosin regulates actin cytoskeleton dynamics. It stabilizes the actin filament and protects it against the actions of DNase I (Hitchcock et al., 1976), cofilin (Bernstein and Bamburg, 1982; DesMarais et al., 2002; Nagaoka et al., 1995; Nakano and Mabuchi, 2006; Nishida et al., 1984; Ono and Ono, 2002), and gelsolin (Pruliere et al., 1986). It inhibits Arp2/3 complex nucleation of branched actin filaments (Blanchoin et al., 2001). Tropomyosin protects the pointed, slow-growing end of the filament, alone (Broschat, 1990; Broschat et al., 1989) and with tropomodulin in vertebrate cells (Colpan et al., 2013; Kostyukova and Hitchcock-DeGregori, 2004; Kostyukova et al., 2005; Weber et al., 1994; Yamashiro et al., 2012). Tropomyosin is a positive regulator of formin function at the barbed, fast-growing end of the filament (Skau et al., 2009; Ujfalusi et al., 2009, 2012; Wawro et al., 2007) and competes with and inhibits actin crosslinking proteins, including α-actinin, filamin, and fimbrin (Abe and Maruyama, 1973; Drabikowski and Nowak, 1968; Maruyama and Ohashi, 1978; Skau et al., 2011; Skau and Kovar, 2010; Zeece et al., 1979). Tropomyosin regulates motility by making the interaction of myosin with actin positively- or negatively-cooperative, depending on the myosin and tropomyosin isoforms (Barua et al., 2014; Bremel and Weber, 1972; Clayton et al., 2010, 2012, 2015; Fanning et al., 1994; Hodges et al., 2012; Lehrer and Morris, 1982; Stark et al., 2010; Tang and Ostap, 2001). We reached this understanding of tropomyosin regulatory functions primarily based on *in vitro* experiments. Translation to an *in vivo* venue has been challenging because of the redundancy of the animal proteome. The presence of four genes encoding more than 40 isoforms in mammals (Geeves et al., 2014) makes genetic and cellular studies in vertebrates, as well as invertebrates, a challenge.

While the cytoskeletal proteins that have been investigated in yeast have homologs in most eukaryotes, tropomyosin has been identified only in animals and fungi (Ophisthokonts), but not plants, amoebae, slime molds or other protists (Barua et al., 2011; Cranz-Mileva et al., 2013). Budding yeast has two tropomyosin genes, TPM1 and TPM2 (Drees et al., 1995; Liu and Bretscher, 1989). Disruption of TPM1 results in loss of actin cables and interruptions in the secretory pathway (Liu and Bretscher, 1989, 1992). Disruption of TPM2 has no detectable phenotype but is lethal in combination with disruption of TPM1 (Drees et al., 1995).

Fission yeast has a single, essential tropomyosin gene, *cdc8* (Balasubramanian et al., 1992). Disruption of the gene to create a null mutant results in the absence of actin cables, depolarization of actin patches, inability to form the actin-containing contractile ring leading to failure of cytokinesis (Balasubramanian et al., 1992), and inability to form mating tubes for fusion (no zygote formation) (Kurahashi et al., 2002). Essentially the same phenotype is observed in *cdc8^ts^* mutants at the restrictive temperature (Chang et al., 1996). Tropomyosin is required for contractile ring integrity during contraction (Mishra et al., 2013). Extensive analysis of the function of actin dynamics and assembly of actin-containing structures in fission yeast provides the context for *in vivo* study of structure-function relationships in tropomyosin (Kovar et al., 2011).

The fission yeast cytoskeleton uses mechanisms that are conserved in most eukaryotes for processes that include cytokinesis, intracellular transport, and establishment of cellular polarity (Mishra et al., 2014). *Schizosaccharomyces pombe* has emerged as a model organism for study of these processes because its simpler genome encodes a smaller and less redundant proteome than in mammals, the facility of genetic manipulation, and the amenability of the cytoskeleton to microscopic study in living cells.

For these reasons we directed our attention to development of an evolution-based molecular-genetic approach of functional analysis in fission yeast as a way to dissect the molecular basis of known and unknown functions of tropomyosin in a living cell. The overarching hypothesis is that residues required for conserved tropomyosin functions are conserved. The approach follows from our evolutionary analysis of mammalian tropomyosins using *in vitro* functional assays of conserved functions including actin binding and myosin regulation (Barua et al., 2011, 2012, 2013, 2014).

In earlier work (Cranz-Mileva et al., 2013) we identified the evolutionarily-conserved codons in fungal tropomyosins, and we screened a series of *cdc8* Ala or Thr mutations at conserved sites on the coiled coil surface for the ability to rescue the growth and cellular phenotype of a *cdc8^ts^* mutant at the restrictive temperature. While all rescued growth, certain mutations affected actin cable organization, contractile ring formation, actin patch polarization and cellular shape. We selected sites of interest and created three gene replacement strains carrying mutations at two or three sites in the *cdc8* gene. All three strains were isolated as diploids and the mutations severely reduced the *in vitro* affinity of recombinant tropomyosin for filamentous actin in two of the three, limiting interpretation of the results.

Here we analyze a series of gene replacement mutants that are viable as haploids, and studied the effects of the mutations on actin affinity and organization of the actin cytoskeleton and associated proteins. While all the mutants are able to divide (since they are viable) and mate, the mutations result in one or more of the following cytoskeletal phenotypes: altered actin cable morphology, abnormal or incomplete actin contractile ring assembly, depolarization of actin patches, and deficiencies in vacuole fusion. The type of cytoskeleton alteration depends on the mutation, indicating that specific functions depend on particular tropomyosin residues, some of which are in putative actin binding sites based on homology with mammalian tropomyosins. When *cdc8* mutants were crossed with strains expressing cytoskeleton proteins with fluorescent protein tags, in some cases synthetic effects were observed, inferring interaction of the mutated site(s) on *cdc8* with the cytoskeletal protein.

## RESULTS

### A cdc8 mutation that inhibits assembly of actin cytoskeletal structures enables localization of early contractile ring components

The most severe mutant in our proteomic screen of the effect of mutations at conserved sites in *cdc8* on cellular morphology and function was *cdc8^R121A.D131A.E138A^* (Cranz-Mileva et al., 2013). It was the least effective mutant, among those studied, in rescuing a temperature-sensitive mutant (*cdc8-27*) at the restrictive temperature. The cell shape was abnormal, the actin cables, contractile ring and septum were poorly organized, and there was irregular polarization of actin patches. A gene replacement strain, *cdc8^R121A.D131A.E138A^*, isolated as a homozygous diploid, had many of the same cellular features. However, the mutant maintained some cellular function compared to a temperature-sensitive mutant that was unable to assemble any actin cytoskeletal structures or to divide at the restrictive temperature. The *in vitro* actin affinity of recombinant Cdc8p^R121A.D131A.E138A^ was too weak to measure.

**Fig. 1.**
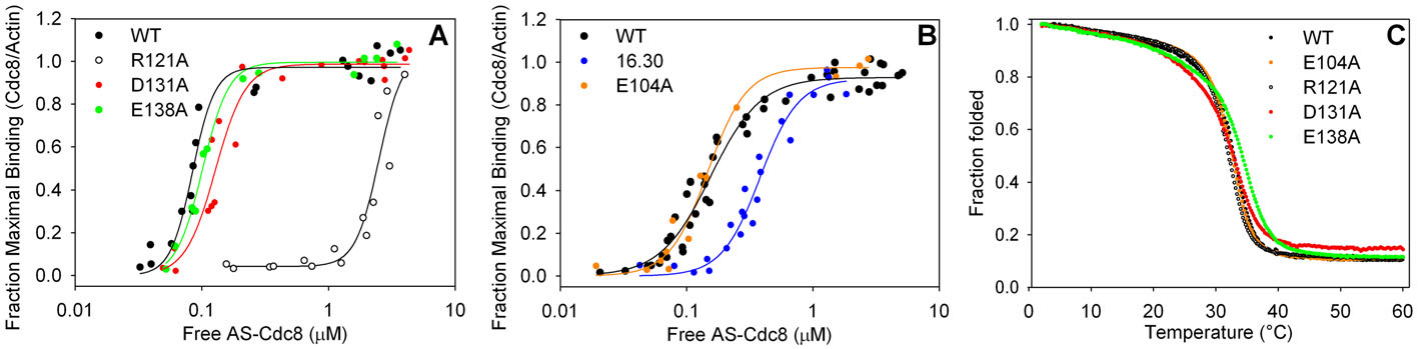
**Actin affinity and thermal stability of wildtype and mutant fission yeast tropomyosins.** (A,B) Actin affinity of AS-Cdc8p, wildtype and mutants, measured by cosedimentation as described in Materials and Methods (20 mM MOPS pH 7.0, 150 mM NaCl, 2 mM MgCl_2_, 5 µM actin). (A) AS-Cdc8p^wt^, K_app_=11.8×10^6^ M^−1^ (*n*=3); AS-Cdc8p^R121A^, K_app_=0.40×10^6^ M^−1^ (*n*=2); AS-Cdc8p^D131A^, K_app_=7.8×10^6^ M^−1^ (*n*=3); AS-Cdc8p^E138A^, K_app_=9.9×10^6^ M^−1^ (*n*=2). (B) AS-Cdc8p^wt^, K_app_=6.6×10^6^ M^−1^ (*n*=2); AS-Cdc8p^D16A.L30A^, K_app_=2.6×10^6^ M^−1^ (*n*=3); AS-Cdc8p^E104A^, K_app_=6.8×10^6^ M^−1^ (*n*=2). The binding experiments in A and B were done at different times with different actin preparations. The data for AS-Cdc8p^D16A.L30A^ are reproduced from (Cranz-Mileva et al., 2013) in which the wildtype K_app_=6.3×10^6^ M^−1^ (*n*=3). (C) Thermal stability determined by measuring the ellipticity at 222 nm between 0–60°C. The ellipticity at 2°C is normalized to 1. The melting temperature (T_M_) is defined as the temperature where the normalized ellipticity is 0.5. The observed T_M_ (*n*=1) are: AS-Cdc8p^wt^=33°C, AS-Cdc8p^D16A.L30A^=34°C (Cranz-Mileva et al., 2013); AS-Cdc8p^E104A^=33°C, AS-Cdc8p^R121A^=32°C, AS-Cdc8p^D131A^=33.0°, AS-Cdc8p^E138A^=34°.

In order to identify the contributions of the mutations at individual residues mutated in *cdc8^R121A.D131A.E138A^* to tropomyosin function, we made gene replacement strains with single site mutations (*cdc8^R121A^* [SH39, SH40], *cdc8^D131A^* [SH43], *cdc8^E138A^* [SH34]; supplementary material Table S1) and expressed recombinant protein in *E. coli* with the single site mutations (Cdc8p^R121A^, Cdc8p^D131A^, Cdc8p^E138A^). All three mutants were isolated as haploid strains and have normal growth parameters with the exception of *cdc8^R121A^* that could not be accurately measured (supplementary material Fig. S1). The recombinant proteins were expressed with the N-acetylation mimic, AlaSer, at the N terminus (Monteiro et al., 1994) since unacetylated Cdc8p binds poorly to actin (Cranz-Mileva et al., 2013; Maytum et al., 2000). Cdc8p^R121A^ binds actin with ∼30-fold weaker affinity than wildtype, and Cdc8p^D131A^ and Cdc8p^E138A^ have close to wildtype affinity (Fig. 1A). The effect of the mutations on stability is minimal (Fig. 1C).

**Fig. 2.**
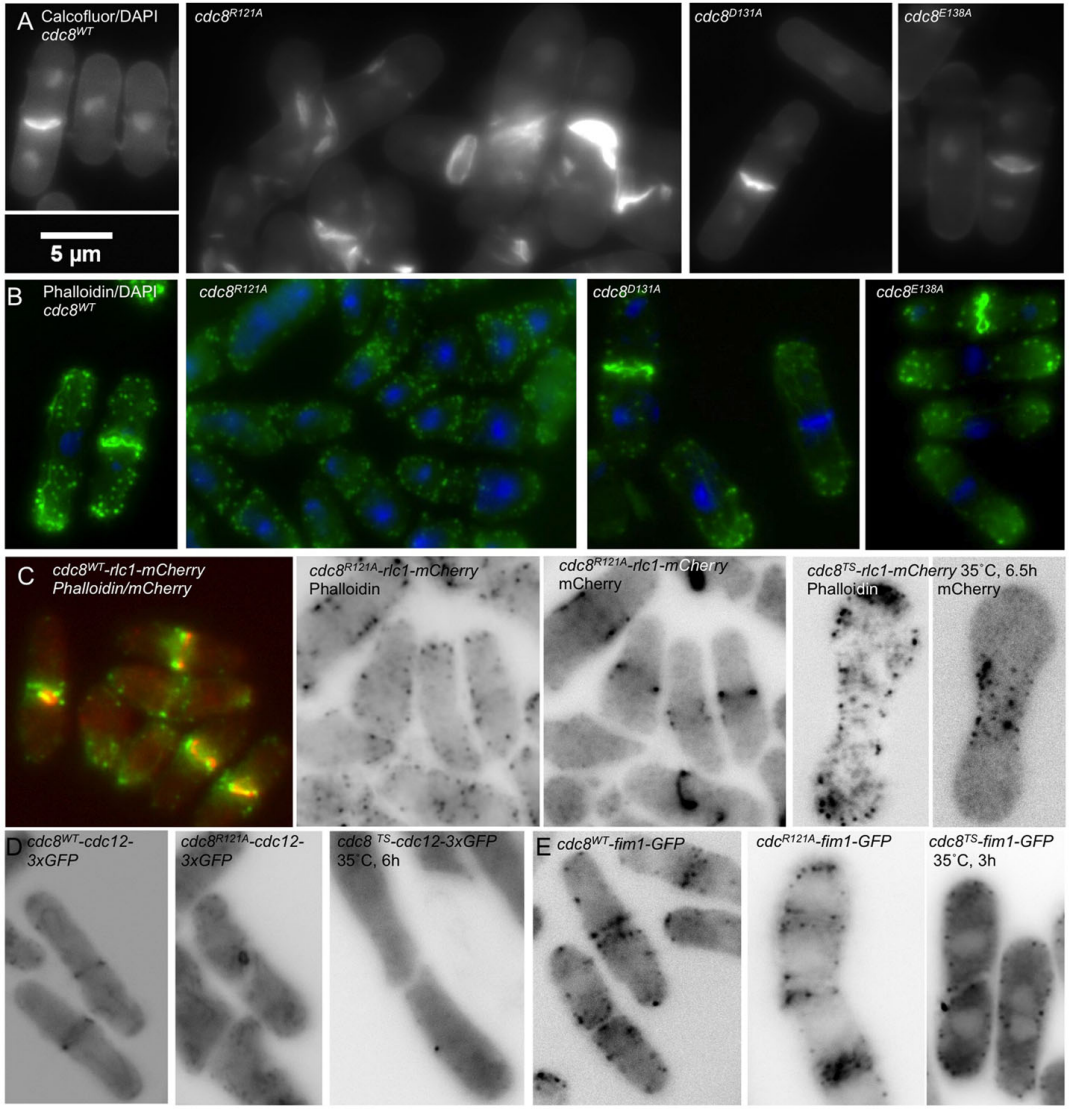
**Morphology and cytoskeleton organization of cdc8^wt^, cdc8^R121A^, cdc8^D131A^, cdc8^E138A^.** (A) Fixed wildtype (SH30) and mutant cells stained with Calcofluor to show septum and septal material and DAPI to show nuclei. The *cdc8^R121A^* cells (SH39) had disorganized septa and abnormal cell shapes. Cells with up to four nuclei were observed. *cdc8^D131A^* (SH43) and *cdc8^E138A^* (SH34) appeared normal. (B) Staining of the actin cytoskeleton using Alexa-phalloidin (green), and nuclei with DAPI (blue) showed dispersed patches, the absence of actin cables and poorly condensed actin rings in *cdc8^R121A^*. The other strains appeared normal. (C) *cdc8^wt^-rlc1-mCherry* (SH49) and *cdc8^R121A^-rlc1-mCherry* (SH52) cells fixed and stained with Alexa-phalloidin (green) to visualize the actin cytoskeleton. m-Cherry (red) shows localization of myosin II in the contractile ring in *cdc8^wt^*. In *cdc8^R121A^-rlc1-mCherry* myosin II assembles into contractile rings despite the absence of organized actin in contractile rings or cables. For comparison, in *cdc8-27-rlc1-mCherry* (SH81), a temperature-sensitive mutant, neither the actin (Alexa phalloidin) nor myosin II (*rlc1-mCherry)* assembled into a contractile ring at the restrictive temperature (35°C). Other cells were grown and fixed at 30°C. (D) Formin-GFP (Cdc12p-GFP) fluoresence. Formin localized in rings in wildtype (SH57) but, like Rlc1p-mCherry, formin (Cdc12p) assembled into abnormal rings in *cdc8^R121A^* (SH60) (live cells). No midline localization was observed in a temperature sensitive strain (SH87) at the restrictive temperature (fixed cells at 35°C). (E) Fimbrin-GFP fluorescence (live cells confocal image). Fim1p-GFP localized in the midline region in *cdc8^wt^* (SH77) and *cdc8^R121A^* cells (SH75) (live cells), but not in the temperature-sensitive strain (SH83) at the restrictive temperature (cells grown and fixed at 35°C). The localization of fluorescent proteins was normal in *cdc8^D131A^* and *cdc8^E138A^* strains (supplementary material Fig. S3).

Of the three strains, the actin cytoskeleton is severely affected in *cdc8^R121A^*, while the D131A and E138A mutations have little effect. Fig. 2 shows the septum with Calcofluor, nuclei with DAPI (Fig. 2A) and actin cytoskeleton visualized with Alexa-phalloidin (Fig. 2B). While the actin cytoskeleton and septa are apparently normal in *cdc8^D131A^* and *cdc8^E138A^*, these features are disrupted in *cdc8^R121A^* resulting in the near absence of actin cables and dispersed actin patches. Alexa-phalloidin staining in the midline region indicates the presence of actin filaments, but they do not condense into contractile rings (Fig. 2B,C). Septal material accumulates but does not form a discrete septum (Fig. 2A). Tropomyosin is expressed in *cdc8^R121A^* but it is localized in patches rather than the contractile ring and cables as in wildtype or other mutants (supplementary material Fig. S2). Even though the effect of the R121A mutation on cellular morphology is severe, some cells can divide as evidenced by isolation and maintenance of a viable haploid strain.

Since *cdc8^R121A^* can divide without formation of a discrete actin-containing contractile ring, we investigated the localization of proteins that are found in the area of the ring and known to be regulated by tropomyosin in fission yeast and/or mammalian cells: myosin II, fimbrin, and formin (Cdc12p) (Kovar et al., 2011). We created wildtype and mutant *cdc8* strains expressing Rlc1p-mCherry (regulatory light chain of myosin II [Myo2p, Myp2p], SH49 *cdc8^wt^*, SH52 *cdc8^R121A^*), Fim1p-mEGFP (fimbrin, SH77 *cdc8^wt^*, SH75 *cdc8^R121A^*), Cdc12p-3xGFP (formin, SH57 *cdc8^wt^*, SH60 *cdc8^R121A^*) and Act1-LifeAct-GFPp, a live cell marker for filamentous actin (Riedl et al., 2008) (SH136, *cdc8^R121A^*).

We were surprised that in *cdc8^R121A^* Rlc1p-mCherry localizes to form an organized but misshapen contractile ring that closes when the cell divides (Fig. 2C). Cdc12p-3xGFP, if observed, appears to form a contracted but asymmetrically-localized ring, reminiscent of a ring that contracted without being attached to the plasma membrane (Fig. 2D). Finally, Fim1p-mEGFP localizes in the midline area in a cortical pattern that is patchy rather than ring-like (Fig. 2E). The R121A mutant retains some function compared to a *cdc8-27*, a temperature-sensitive mutant that is unable to initiate the first stages of contractile ring assembly at the restrictive temperature: Rlc1p-mCherry and Fim1p-mEGFP have no evident localization at the restrictive temperature (Fig. 2C,E) and Cdc12p-3xGFP is seen as a single dot (Fig. 2D). The *cdc8^R121A^* strain has an apparent mating deficiency with *ain1-mEGFP* (JW1144). In several attempts asci formed that were filled with a single dark mass, but discrete spores were never seen, indicative of a synthetic negative effect of the *cdc8* R121A mutation and GFP modification of α-actinin.

Mirroring the wildtype-like appearance of the actin cytoskeleton in *cdc8^D131A^*and *cdc8^E138A^* cells, strains expressing Rlc1p-mCherry (SH54 *cdc8^D131A^*, SH56 *cdc8^E138A^*), Fim1p-mEGFP (SH74 *cdc8^D131A^*, SH75 *cdc8^E138A^*) and Cdc12p-3xGFP (SH62 *cdc8^D131A^*, SH64 *cdc8^E138A^*), Ain1p-mEGFP (SH76 *cdc8^D131A^*, SH77-A *cdc8^E138A^*) all form normal contractile rings (supplementary material Fig. S3). Normal localization of tropomyosin in the contractile ring and actin cables (supplementary material Fig. S2) and of Myo52p-3xYFP at the poles in both *cdc8^D131A^* and *cdc8^E138A^* (supplementary material Fig. S3) provides further evidence of the apparent neutral effect of the *cdc8* D131A and E138A mutations on these processes. The microtubule cytoskeleton, visualized in *mRFP-atb2* strains is normal for all mutants in our study (supplementary material Fig. S3).

### A conserved residue in fungal tropomyosins is important for polar distribution of actin patches and cell separation at the end of cytokinesis

One of the most highly conserved regions in fungal tropomyosins is the third actin binding period of Cdc8p that includes residues 85–119 (Cranz-Mileva et al., 2013). A residue of particular interest to us is E104, a conserved residue in a surface *c* position in the coiled coil heptapeptide repeat in fungal tropomyosins. The region is less well conserved in period 3 of animal tropomyosins in which the analogous residue is Thr108 in vertebrates, but variable in other animal species (Barua et al., 2011). Therefore, we expressed Cdc8p^E104A^ and constructed *cdc8^E104A^* strains (SH41, SH104, SH105) because of the possibility to identify a fungal-specific function for this conserved residue.

**Fig. 3.**
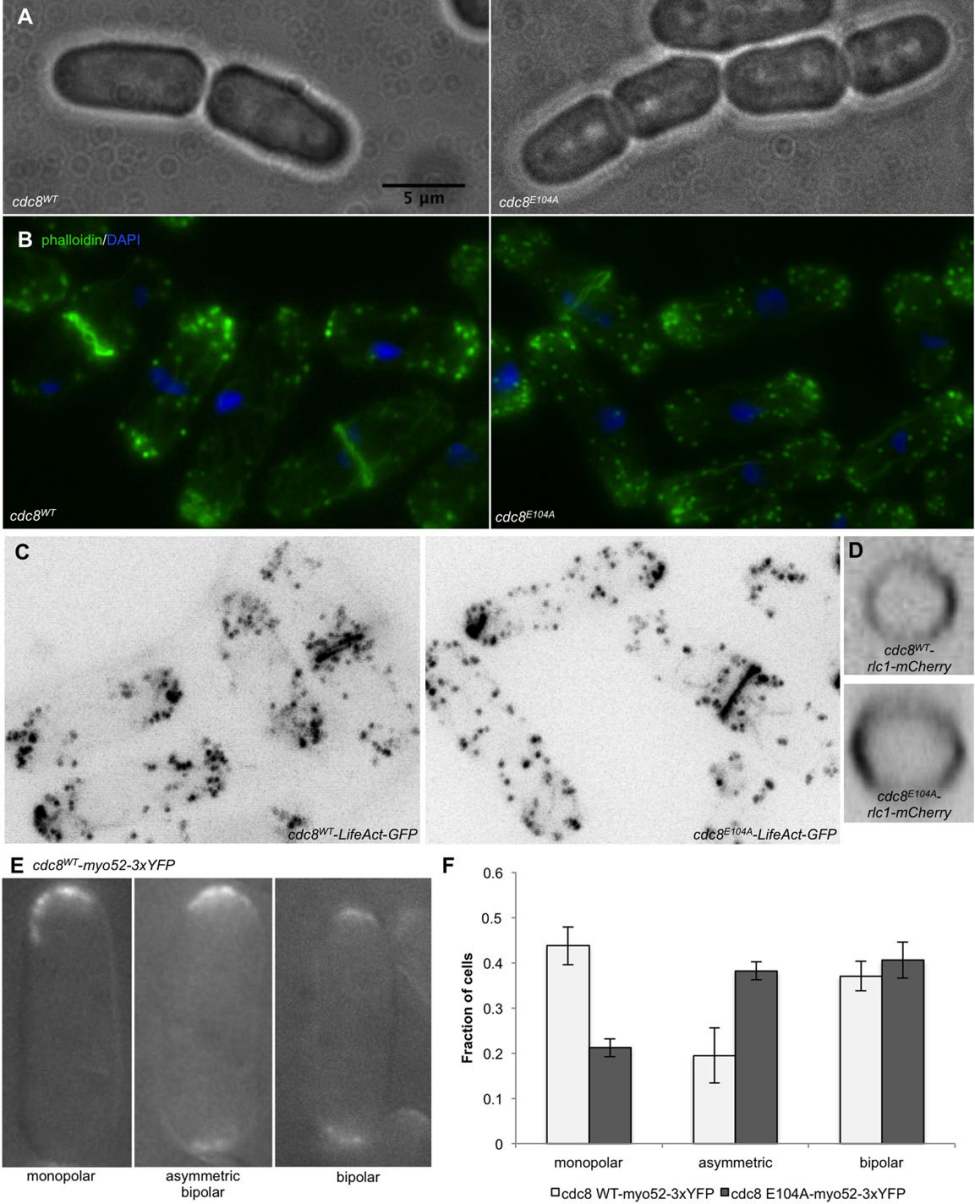
**Cellular morphology and patch distribution in cdc8^E104A^.** (A) Images of live *cdc8^wt^* (SH30) and *cdc8^E104A^* cells (SH41) in phase showing that cells often remain linked after division in *cdc8^E104A^*. The fraction of chains of four cells linked together was measured for wildtype (0.0055±0.013) and *cdc8^E104A^* (0.069±0.025) from 3 independent experiments of *n*>270 (*P*=0.0007). (B) Wildtype and mutant cells fixed and stained with Alexa-phalloidin (green) and DAPI (blue) to visualize F-actin and DNA. The actin patches are more dispersed in mutant than in wildtype cells, but the cables and contractile rings are similar to wildtype. (C) Confocal images of representative fields of live *cdc8^wt^*-*LifeAct-GFP* (KV587) and *cdc8^E104A^*-*LifeAct-GFP* cells (SH134) illustrate apparently normal cables and contractile rings in the mutant, but patches are less tightly polarized to the poles. The patch distribution was quantified by dividing individual cells lengthwise into three zones, two polar zones each representing 12.5% of the total length and a central zone representing 75% of the total length. The fluorescence intensity in each zone was quantified and corrected for background using ImageJ. The fraction of the total fluorescence in the polar zones was calculated: wildtype (0.46±0.08) and *cdc8^E104A^* (0.38±0.06) (*n*=26, *P*=0.00025). (D) Cross-sections of 2D reconstructions of live *cdc8^wt^*-*rlc1-mCherry* (SH49) and *cdc8^E104A^*-*rlc1-mCherry* cells (SH130) showing normal myosin II rings. (E) *cdc8^E104A^-myo52-3xYFP* cells (SH98) illustrating monopolar, asymmetric and bipolar patch distribution. (F) Fraction of *cdc8^wt^* (SH107) and *cdc8^E104A^* cells (SH98) with each distribution based on fluorescence intensity quantification from three independent measurements, each *n*>70 cells. The fluorescence at each cell tip was quantified and corrected for background using ImageJ. The ratio of fluorescence at one end to the fluorescence at the other end was measured. If the ratio was ≤0.33 the cell was “monopolar”, >0.33 but <0.66, the cell was “asymmetric bipolar”, or ≥0.66 the cell was “bipolar”. The fraction of bipolar cells was similar in wildtype (0.37±0.03) compared to mutant (0.41±0.04). *cdc8^E104A^* had a decreased fraction of monopolar cells (0.21±0.02) and an increased fraction of asymmetric bipolar cells (0.38±0.02) compared to wildtype cells (monopolar, 0.44±0.04; asymmetric bipolar, 0.20±0.06). In these experiments, the lengths of monopolar, asymmetric bipolar, and bipolar cells were also measured in *cdc8^wt^*-*myo52-3xYFP* (9.0±0.8 µm, 9.6±1.4 µm, 11.0±1.4 µm) and *cdc8^E104A^-myo52-3xYFP* (9.6±1.3 µm, 9.5±0.8 µm, 10.4±1.4 µm). Error bars show mean±standard deviation (s.d.).

The *cdc8^E104A^* strain exhibits a prominent cellular phenotype in live cultures in which there is an increased fraction of four cells linked at the ends, as in sausage links (Fig. 3A; *cdc8^wt^*: 0.0055±0.013; *cdc8^E104A^*: 0.069±0.025; *n*>808, *P*=0.0007). The cell shape and size, nuclear number and growth rate are normal (supplementary material Fig. S1). Even though the actin affinity of recombinant Cdc8p^E104A^ and thermal stability are normal (Fig. 1B,C), the actin cytoskeleton is not. The patches are less compact at the poles, more diffuse than in wildtype (Fig. 3B,C). In cells with a bipolar distribution of actin patches 46±0.08% of the total fluorescence is localized at the poles in wildtype, compared to 38±0.06% in *cdc8^E104A^* (*n*=26, *P*=0.0025; details in Fig. 3 legend). The contractile rings and cables appear quite normal, as visualized using Alexa-phalloidin (Fig. 3B, SH41), LifeAct-GFP (Fig. 3C, SH134), or Rlc1p-mCherry (myosin II, Fig. 3D, SH130), consistent with an apparently normal tropomyosin localization (supplementary material Fig. S2). Therefore we focused on questions related to actin patch distribution and intracellular transport. The localizations of other cytoskeletal proteins with fluorescent protein tags (tubulin (SH133), formin (Cdc12p, SH132), α-actinin (SH95), Myo1p (SH96), coronin (SH128), and Myo52p (SH98) are normal (supplementary material Fig. S3).

To understand how the E104 mutation might affect Cdc8p function, we considered Myo52, a myosin V-class myosin that is involved in vesicular and organelle transport in cells (Hammer and Sellers, 2012). In fission yeast, deletion of *myo52* results in altered polarity of actin patches (Motegi et al., 2001; Win et al., 2001) and altered vacuole fusion and distribution (Mulvihill et al., 2001). The phenotype of poor polarization of patches in *cdc8^E104A^* is shared with *myo52Δ*, suggesting E104 may be involved in regulating Myo52p-dependent processes. *In vitro* studies have shown that Cdc8p increases the affinity of Myo52p for actin, and increases the actin-activated ATPase of Myo52p (Clayton et al., 2010).

To inquire about the Myo52p function in *cdc8^E104A^*, we quantified the localization of Myo52p-3xYFP in a *cdc8^E104A^myo52-3xYFP* strain (SH98). Myo52p-3xYFP is tightly clustered at the cell poles in interphase cells (Fig. 3E) and at the midline of dividing cells. We calculated the ratio of fluorescence intensity at the two ends and categorized the cells according to the polarity of the Myo52p fluorescence distribution. Assuming perfect bipolar distribution would have a ratio of 1.0, we created the following classifications: monopolar (≤0.33), bipolar (≥0.66), or “asymmetric bipolar” (>0.33 but <0.66). An asymmetric bipolar distribution of patches indicates a cell that is between polarities. Even though Myo52p-3xYFP localizes to the poles in *cdc8^E104A^*, the fraction of cells with monopolar localization is less than in wildtype (Fig. 3F, details in legend). Similarly, *cdc8^E104A^* has fewer cells than *cdc8^wt^* cells with a monopolar distribution of actin patches visualized using coronin (Crn1p-GFP) (supplementary material Fig. S4, details in legend). Crn1p-GFP is localized in the patches that are usually coincident with actin, as previously reported (Pelham and Chang, 2001). With both probes *cdc8^E104A^* has more cells with an asymmetric bipolar distribution than *cdc8^wt^* (Fig. 3F, supplementary material Fig. S4). The average length of the asymmetric bipolar cells is <9.5 mm, indicating they are in the pre-NETO stage of the cell cycle (Mitchison and Nurse, 1985).

In addition to vesicle and organelle movement in cells, in fission yeast Myo52 is involved in vacuole fusion (Mulvihill et al., 2001). When Myo52p-3xYFP cells are transferred from YEA medium to water, the Myo52p-3xYFP is redistributed from the cellular poles to around vacuoles within ten minutes. The effect is reversible. We observed no difference between *cdc8^wt^* and *cdc8^E104A^* cells indicating the mutation does not affect endocytosis (supplementary material Fig. S5). However, the *cdc8^E104A^* mutation alters the fusion of vacuoles.

**Fig. 4.**
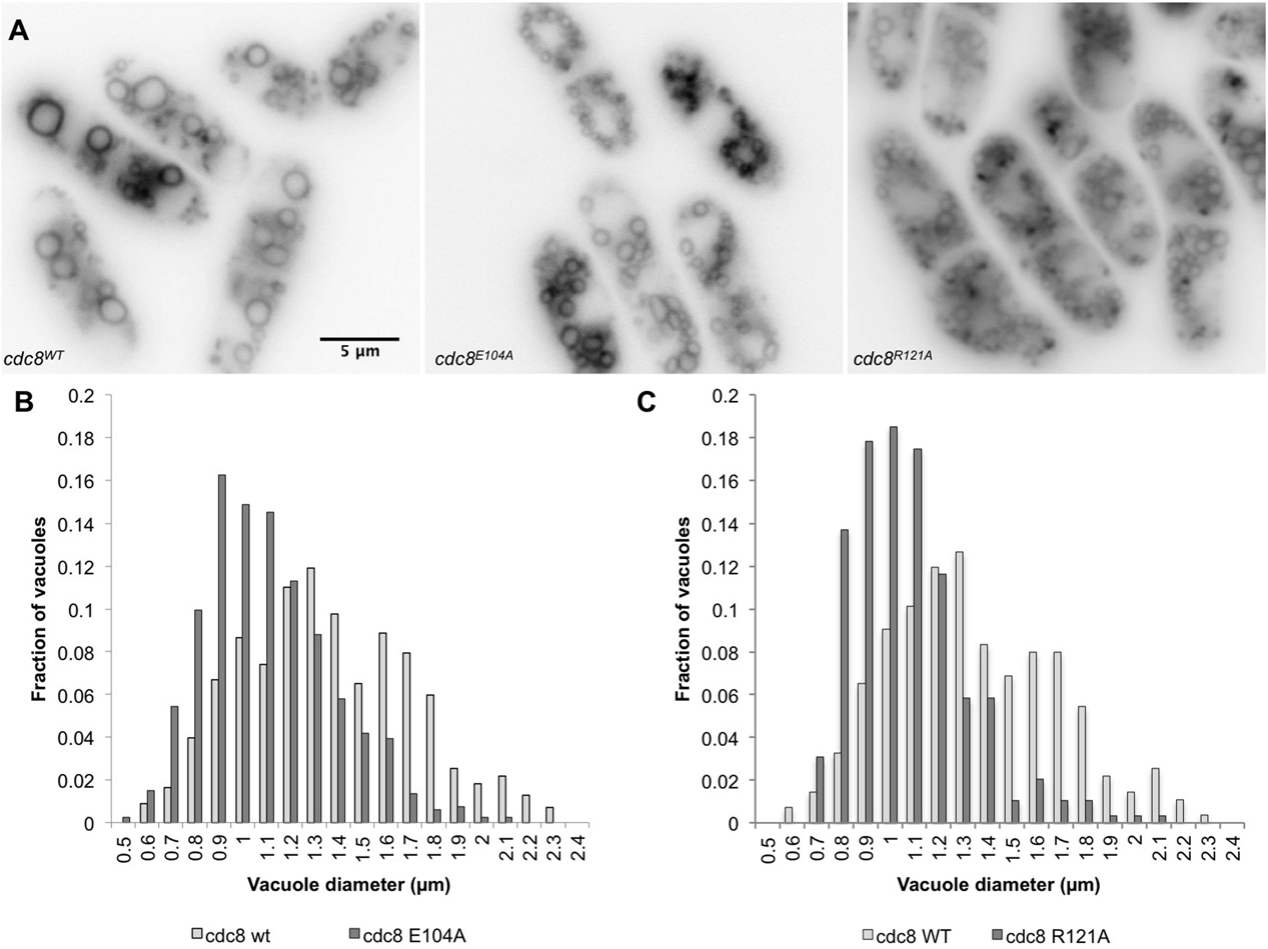
**Analysis of vacuole fusion in cdc8^E104A^ using the lipophilic dye, FM4-64.** Cells were incubated for 45 min. in FM4-64, washed and transferred to medium as described in Materials and Methods. The images are of live cells after 60 min. (A) *cdc8^wt^* (SH30), *cdc8^E104A^* (SH41), *cdc8^R121A^* (SH39). (B) The size distribution of vacuoles in *cdc8^wt^* and *cdc8^E104A^* cells based on two independent experiments, each *n*>140. Mean±s.d.: *cdc8^wt^*, 1.4±0.4 µm; *cdc8^E104A^*, 1.1±0.3 µm (*P*=4.71×10^−31^). (C) The size distribution of vacuoles in *cdc8^wt^* and *cdc8^R121A^* cells in two independent experiments, each *n*>138. Mean±s.d.: *cdc8^wt^*, 1.3±0.3 µm; *cdc8^R121A^*, 1.1±0.2 µm (*P*=5.52×10^−42^). In both mutants the vesicles are smaller than in wildtype but greater in number. The vacuole size distributions in *cdc8^D16A.K30A^*, *cdc8^D131A^*, and *cdc8^E138A^* are indistinguishable from *cdc8^wt^*.

We followed the process of vacuole fusion using the lipophilic dye, FM4-64, that enters vacuoles by endocytosis (Brazer et al., 2000). Whereas the rate of labeling of vacuoles was similar in *cdc8^wt^* and *cdc8^E104A^* cells, the vacuoles are smaller but more numerous in *cdc8^E104A^* cells (*cdc8^wt^*, 6.8±1.9; *cdc8^E104A^*, 10.2±2.9 vacuoles/cell, *n*=48) (Fig. 4). In both *cdc8^wt^* and *cdc8^E104A^* the vacuoles increased in size with time, but they remained consistently smaller in *cdc8^E104^* (Fig. 4B, details in legend). Also, as in *myo52Δ* cells (Mulvihill et al., 2001), the vacuoles tend to cluster around the nucleus (Fig. 4A). We interpret our results to indicate that in *cdc8^E104A^* cells vacuoles form normally but are delayed in fusion, a *myo52*-dependent process.

For comparison, we analyzed vacuole fusion in the other mutants in the present study. The D121A mutation that severely impairs actin affinity and cytoskeletal organization, as described above, results in smaller but more numerous vacuoles (Fig. 4A,C). A mutation that has a major effect on actin cable assembly would be expected to affect *myo52*-dependent processes that involve transport along cables. Alternatively, smaller and more numerous vacuoles may result from vacuole fragmentation in the stressed *cdc8^R121A^* mutant. Three other mutants, *cdc8^D16A,K30A^, cdc8^D131A^, cdc8^E138A^*, have no effect on vacuole fusion (supplementary material Fig. S6). The results lend credence to our postulate that the single E104A mutation affects vacuole fusion through regulation of Myo52p function, a subject for future investigation.

### A synthetic effect of Cdc8p in a strain expressing fimbrin-GFP gives insights into protein function

We previously described *cdc8^D16A.K30A^* (SH22) that we isolated as a diploid strain (Cranz-Mileva et al., 2013). We noted a mild phenotype with occasional less polar distribution of patches and actin cables that tended to be more wavy and reticular than in wildtype. D16 and K30 are conserved surface residues near the N-terminal to C-terminal overlap region of tropomyosin, and D16 is part of a conserved actin binding motif in tropomyosins (Barua et al., 2013, 2011). The respective residues in human tropomyosin, E16 and K30, are among the most conserved in animal tropomyosins (Barua et al., 2011). The actin affinity of recombinant Cdc8p^D16A.K30A^ with an AlaSer N-terminal modification is about 2-fold weaker than wildtype (Cranz-Mileva et al., 2013) (Fig. 1B), whereas a D16A mutation in an unacetylated recombinant protein increased actin affinity (East et al., 2011). A D16A mutation in a mammalian tropomyosin has little effect on the overall actin affinity (Barua et al., 2013). Therefore, we postulate D16 contributes to another conserved tropomyosin function.

**Fig. 5.**
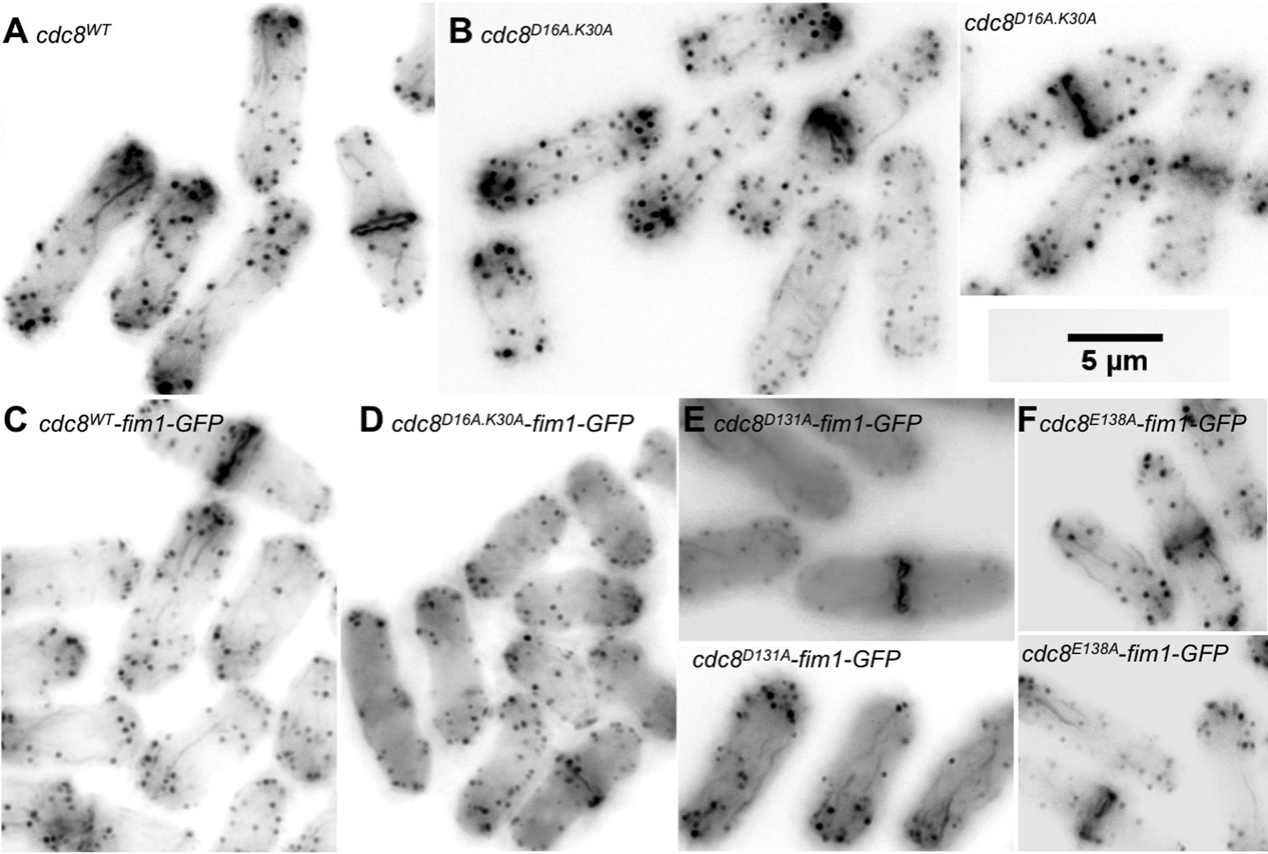
**Actin cytoskeleton in wildtype and mutant cdc8 cells expressing GFP-fimbrin, illustrating synthetic negative effect of cdc8^D16AK30A^ and fim1-GFP.** All cells were fixed and stained with Alexa phallodin. Fim1-GFP fluorescence is weak following fixation. (A) *cdc8^wt^* cells (SH30). (B) *cdc8^D16AK30A^* cells (SH22). (C) *cdc8^wt^* *fim1-GFP* cells (SH77). (D) *cdc8^D16AK30A^ fim1-GFP* cells (SH112). Note the absence of cables. (E) *cdc8^E131A^ fim1-GFP* cells (SH74). (F) *cdc8^E138A^ fim1-GFP* cells (SH75-A). The actin cytoskeleton appears normal in E and F.

In the course of working with the *cdc8^D16A.K30A^* diploid we isolated a spontaneous haploid strain with normal growth parameters (supplementary material Fig. S1) that we analyzed in the present study since it gave us the opportunity to identify the effect of mutating these conserved sites on other cellular functions. We constructed strains of *cdc8^D16A.K30A^* expressing the following tagged cytoskeletal proteins: Rlc1p-mCherry (SH50), Fim1p-mEGFP (SH112), Cdc12p-3xGFP (SH59), Ain1-mEGFPp (SH116) and Act1-LifeActp-GFP (SH122). The distribution of the fluorescent proteins is rather normal in all (supplementary material Fig. S3), as was Cdc8p (supplementary material Fig. S2) and the actin cytoskeleton, except in *cdc8^D16A.K30^fim1-GFP* (Fig. 5). We also note that *cdc8^D16A.K30A^* had difficulty recombining with a *myo1-mGFP* strain (MLY422). While the asci appeared normal, in two crosses only 1/100 colonies screened in random spore analyses had a his^+^ura^+^KanR phenotype. The haploid phenotype was stable during numerous crosses, making the presence of an extragenic suppressor an unlikely explanation, but leaving open the possibility of intragenic suppression.

Fimbrin is an actin filament crosslinking protein found in structures that have bundles of actin filaments, such as filopodia and microvilli in vertebrate cells (Bretscher, 1981; Bretscher and Weber, 1980). In fission yeast, fimbrin is localized in actin patches and in the vicinity of the actin-containing contractile ring in mitotic cells, where it bundles actin filaments and inhibits depolymerization by cofilin (Nakano et al., 2001). Fimbrin is not found in actin cables (Nakano et al., 2001; Wu et al., 2001). In contrast, Cdc8p is predominantly in actin cables and the contractile ring (Balasubramanian et al., 1992). Fimbrin-GFP localization depends on filamentous actin since it is sensitive to latrunculin and does not localize to the contractile ring area in temperature sensitive mutants of various genes, including *cdc8-27,* that do not form F-actin rings at the restrictive temperature (Nakano et al., 2001; Wu et al., 2001). The distribution of fimbrin-GFP in *cdc8^D16A.K30A^* (SH112) is indistinguishable from that in *cdc8^wt^* cells where it was observed, as expected, in patches and in a patchy distribution in the region of the contractile ring (supplementary material Fig. S3). However, whereas the actin cytoskeleton, visualized using Alexa-phalloidin, is apparently normal in *cdc8^wt^-fim1-GFP* cells (SH77) (compare Fig. 5A and C), the *cdc8^D16A.K30A^-fim1-GFP* cells displayed few actin cables (compare Fig. 5B and D). The actin-containing contractile rings are relatively normal, as is actin patch polarization (Fig. 5D). Expression of Fim1p-GFP did not influence the appearance of the actin cytoskeleton of three other mutants described above (*cdc8^D131A^*[SH74], *cdc8^E138A^* [SH75]; Fig. 5E,F) or *cdc8^E104A^* [SH100]. The results infer a synthetic negative effect of Cdc8p^D16A.K30A^ and Fim1p-GFP on cable formation, even though fimbrin is not observed in cables (Nakano et al., 2001; Wu et al., 2001) and present work. These qualitative findings will need to be confirmed using more quantitative methods.

### Effects of LifeAct-GFP on the actin cytoskeleton

**Fig. 6.**
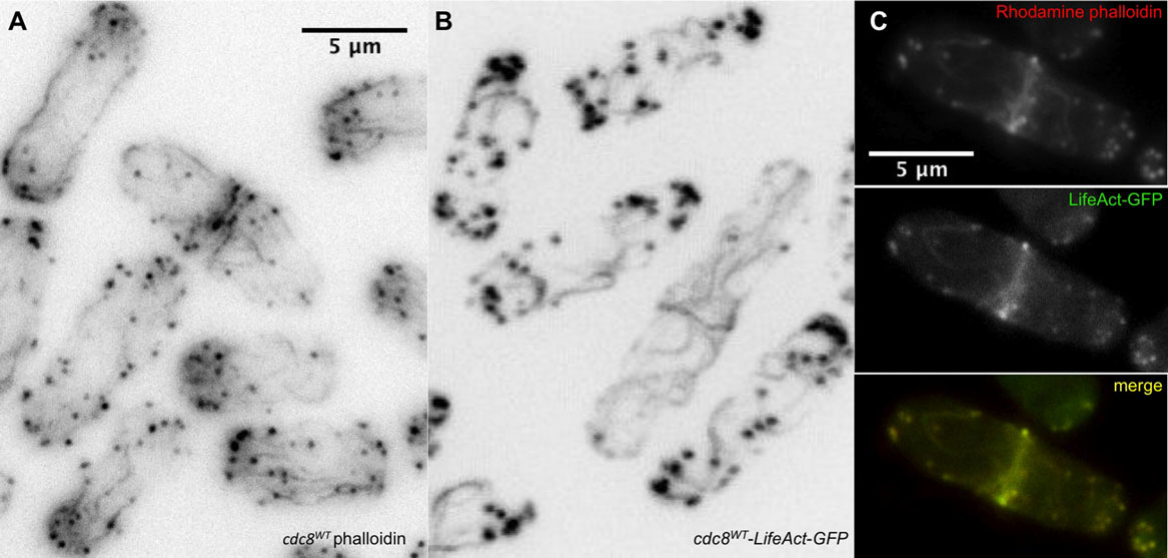
**Comparison of the actin cytoskeleton using Alexa-phalloidin and LifeAct.** (A) *cdc8^wt^* cells (SH30) fixed and stained with Alexa-phalloidin showing long, typically straight actin cables parallel to the long axis of the cells. (B) *cdc8^wt^-LifeAct-GFP* cells (KV587, *ura^−^*) (live cells confocal image) showing wavy cables with variable orientations in relation to the long axis of the cell. The contractile rings are similar in A and B. (C) *cdc8^wt^-LifeAct-GFP* cells (KV587), fixed and stained with rhodamine-phalloidin (left, red channel), green channel showing GFP (center), and superimposed (right). The rhodamine and GFP images superimpose, showing that the phalloidin binds to the same structures that are labeled with GFP.

LifeAct is a 17-amino acid peptide reported to bind both G-actin and F-actin that is used as a probe for F-actin in living cells (Riedl et al., 2008). Localization of LifeAct-GFP is almost identical to that of phalloidin in fixed cells from different species, including fission yeast (Huang et al., 2012; Lemieux et al., 2014) and the present work (Fig. 6C). The synthetic peptide (no GFP) has little effect on actin polymerization *in vitro* (Riedl et al., 2008). For these reasons LifeAct is currently viewed as the best marker for F-actin in living cells.

In our qualitative analysis, however, the actin cables in strains expressing LifeAct-GFP are altered in that they are less straight (Fig. 6A,B). When wildtype *cdc8* cells expressing LifeAct-GFP (KV587) are fixed, the waviness of the cables is similar to those in living cells when visualized using the LifeAct-GFP fluorescence, or when fixed and stained with phalloidin. The LifeAct and phalloidin fluorescence are coincident (Fig. 6C). Therefore, we suggest that LifeAct affects the organization of the actin cytoskeleton in fission yeast even though it has no marked effect on the mating or growth. Similar reservations have been expressed about the use of LifeAct-mEGFP in *Drosophila* where strong overexpression resulted in sterility and defects in the actin cytoskeleton (Spracklen et al., 2014).

## DISCUSSION

In the present work we applied an evolution-based proteomic approach towards understanding tropomyosin function in living fission yeast by creating several *cdc8* mutants that have one or more Ala mutations in conserved surface residues. We found that the mutations have different effects, indicative of residue-specific functions. By combining analysis of the actin cytoskeleton with that of other proteins known to interact with Cdc8p from *in vitro* or cellular studies, we have inferred functional interactions that could be investigated in future studies.

### Discussion of cdc8^R121A^

Our results show that Arg121 has a crucial role for tropomyosin function in fission yeast, primarily due to its involvement in binding actin. R121 is in a position homologous to conserved surface basic residues in mammalian tropomyosins that are part of the actin binding sites on tropomyosin in models based on experimental and molecular dynamics studies (Barua et al., 2013; Brown et al., 2005; Li et al., 2011; Zheng et al., 2013). The reduced actin affinity of Cdc8p^R121A^ argues for a model in which the binding sites of tropomyosin for the highly-conserved protein actin are conserved in animal and fungal proteins. The finding also supports the hypothesis for universality in the functions of other cytoskeletal proteins and their interactions. Since a function shared by most tropomyosins is to stabilize actin filaments, it follows that actin cables and a condensed contractile ring do not form when tropomyosin binds poorly. It is possible that short, unstable actin filaments are present since there is sometimes diffuse or speckled phalloidin staining, versus higher-order assemblies of filaments found in cables and the contractile ring. Clearly, some tropomyosin and associated actin functions remain since *cdc8^R121A^* is viable, unlike the *cdc8* disruption mutant (Balasubramanian et al., 1992) or *cdc8^ts^* mutants at the restrictive temperature (references cited in Cranz-Mileva et al., 2013).

The order of assembly of certain of the contractile ring components has been established (Wu et al., 2003). During the first stage Myo2p and its associated light chains and Cdc12p (formin) assemble in nodes in the contractile ring area (Wu et al., 2003). Actin filaments follow, forming a dense network around the equator as Myo2p and its associated light chains begin to form an equatorial ring (Vavylonis et al., 2008). Laporte et al. (2012) suggest that α-actinin and fimbrin together with Myo2p activity coalesce actin filaments that connect the nodes to form an organized contractile ring. While actin filaments are present in the area of the assembling contractile ring in *cdc8^R121A^*, as evidenced by diffuse fluorescence seen with phalloidin staining and LifeAct-GFP, coalescence of the actin into a ring is not supported. The weaker actin affinity of Cdc8p^R121A^ may make actin filaments more susceptible to severing by cofilin, impair formin (Cdc12p) nucleation of actin filaments for formation of the ring and filaments that are crosslinked by α-actinin or fimbrin, or impair Myo2p function. Our observations support findings that the myosin molecules remain in the ring as it closes, while the actin and associated proteins do not (Mishra et al., 2013).

### Discussion of cdc8^E104A^

Mutation of the conserved surface residue, Glu104 to Ala, has little effect on actin affinity *in vitro*, but it does influence the distribution of actin patches and vacuole fusion even though the cell shape, septum formation and growth are normal. The results indicate a function for *cdc8* in establishing the polarity of the cellular actin cytoskeleton. We suggest this may take place in the following way.

The *cdc8^E104A^* cells remain weakly attached following cytokinesis when viewed in living cells (Fig. 3A). The handling of cells for microscopy involves centrifugation, pipetting and gentle vortexing that breaks the attachment. The asymmetric bipolar distribution of actin patch-associated proteins, including LifeAct-GFP, Myo52p-YFP and Crn1p-GFP may reflect the prior residual end-to-end attachments.

We suggest the observed phenomena of cell-cell links and altered patch distribution reflect a delay in the completion of cell division and redistribution of actin patches following cytokinesis from the new end (site of cytokinesis) to the old end. We never observed chains with more than four cells, suggesting that the cells separate when the “new end” after the first division becomes the “old end” at the next cell cycle. In *cdc8^E104A^* new end take off (NETO) appears to be normal, since the position of the end-most division scar to the new tip of the cell in relation to the cell length (Mitchison and Nurse, 1985) is the same as in *cdc8^wt^*. Also, the fraction of cells with a bipolar distribution of patches is normal. These findings, as well as the observation that *cdc8^E104A^* cells with an asymmetric bipolar patch distribution are pre-NETO (<9.5 µm) suggest that the problem with patch distribution and cell separation occurs during the restoration of monopolarity after cell division. Our results are consistent with the model that one mechanism of transport of the vesicles to the old end involves transport on actin filaments via the plus-end directed myosin, Myo52p, which is regulated by Cdc8p (Clayton et al., 2010). The altered vesicle fusion in *cdc8^E104A^* cells further supports our conclusion. We note that Glu104 is close to a region of muscle tropomyosin that is critical for regulating skeletal muscle myosin (Barua et al., 2014).

### Discussion of cdc8^D16A.K30A^

Fimbrin and tropomyosin bind actin in different manners, yet they both stabilize filaments from severing by cofilin (Adf1p) (DesMarais et al., 2002; Nakano et al., 2001). Cdc8p and fimbrin compete with each other for binding actin *in vitro* (Skau and Kovar, 2010), and *in vivo* as inferred from cellular studies. For example, a *fim1* deletion mutant is not lethal, but the actin patches contain more Cdc8p than in wildtype (Skau and Kovar, 2010). Fimbrin and tropomyosin have opposing effects on myosin 1 at actin patches (Clayton et al., 2010).

The synthetic effect of *cdc8^D16A.K30A^* and *fim1-GFP* in which we observe loss of cables in the *cdc8^D16A.K30A^-fim1-GFP* strain suggests a functional relationship between Cdc8p residues D16A and K30A and Fim1p with regard to cable assembly. Actin filament function in other *cdc8^D16A.K30A^* strains is not obviously affected: patch distribution and Fim1p-GFP localization appear normal. α-actinin, another actin crosslinking protein, is localized in the contractile ring, as in wildtype. Furthermore, the cells divide and mate. A straightforward explanation is that the suboptimal function of Fim1p and Cdc8p, separately, in their mutated or tagged state barely affects cable formation, but when both Fim1p and Cdc8p are compromised we observe a near total loss of cables. In this view, the functions of Cdc8p^D16A.K30A^ and Fim1p-GFP are synergistic and may make the actin filaments more susceptible to depolymerization or severing by one or more actin binding proteins, such as cofilin-Adf1p. An interpretation more consistent with our findings is that Cdc8p^D16AK30A^ competes less effectively for actin with Fim1p-GFP than Fim1p and more filaments are incorporated into actin patches as enabled by the Fim1p-GFP, at the expense of actin cables.

### Conclusions

Our work validates an evolution-based approach as a way to identify new functions for tropomyosin and the sites of potential interactions with other cytoskeletal proteins. Analysis of the effects of site-specific mutations at conserved surface residues in fission yeast tropomyosin indicates the involvement of conserved residues in specific functions. These include actin binding, regulation of the assembly of actin-containing structures that influence the polarity of the actin cytoskeleton, contractile ring assembly, localization of other proteins in the actin cytoskeleton, and cell division.

More specifically, (1) a *cdc8* mutation that has little effect on *in vitro* actin affinity affects patch polarization and as well as vacuole fusion, processes that depend on Myo52p, a member of the myosin V class. (2) A mutation that alone has a mild phenotype is synergistic with GFP-fimbrin, inferring involvement of the mutated tropomyosin sites in interaction between the two proteins. (3) Additional negative synthetic effects were identified by difficulties in mating certain mutants with strains expressing EGFP-α-actinin and Myo1p-mGFP. There may be additional synthetic negative effects that we did not reveal. (4) Proteins known to assemble in the region of the contractile ring before actin (Wu et al., 2003) continue to do so in a mutant *cdc8* strain that fails to assemble a condensed actin ring, providing support for the contractile ring assembly pathway. (5) Of more general significance is the negative effect of the widely-used actin filament probe, LifeAct-GFP, on the actin cytoskeleton in wildtype cells, indicating caution in using this tool as a biomarker for actin filaments.

## MATERIALS AND METHODS

### Schizosaccharomyces pombe strains, plasmid and genetic methods

#### Yeast growth and general methods

Strains were grown in EMM (Edinburgh minimal medium, Sunrise Sciences, San Diego, CA) with the appropriate selective supplements (0.0225% adenine, lysine, histidine, uracil) or YEA medium (0.5% yeast extract, 3% dextrose, 0.015% adenine) following standard growth, genetic and cell biology protocols in (Forsburg, 2003a,b) and other online resources. The strains used and created in this study are listed in supplementary material Table S1. The standard growth temperature was 30°C, 35°C was the restrictive temperature for temperature-sensitive strains, and 25°C was used for permissive growth of temperature-sensitive strains and for other selected procedures. Cells were counted using a hemocytometer. Cellular transformation was carried out using the lithium acetate method (Kanter-Smoler et al., 1994) and purified plasmid or PCR-generated fragments. Genomic DNA was purified according to (Sambrook and Russell, 2001) with modifications. The breaking buffer was 2% Triton X-100, 1% SDS, 100 mM NaCl, 10 mM TrisHCl pH 8.0, 1 mM EDTA; we included an additional chloroform extraction, and RNase A digestion (10 µg/µl, 30 min at 37°C) in the final step. The *cdc8* sequence was verified in the *cdc8* strains and plasmids created in this study using appropriate primers (Genescript, Piscataway, NJ, USA or Genewiz, South Plainfield, NJ, USA).

#### Marker reconstitution mutagenesis

To make gene replacements of the *cdc8^+^* with selected mutations, we used the method developed by Tang et al. (2011) with strains and plasmids previously described (Cranz-Mileva et al., 2013).

The plasmid pH5c-*cdc8^+^* (Cranz-Mileva et al., 2013) is the template for introducing mutations into *cdc8.* The mutations were made by Mutagenex (Hillsborough, NJ, USA) and verified by DNA sequencing (Genescript or Genewiz). PCR fragments were amplified as previously described and transfected into SH13. Transformants were selected on minimal medium lacking histidine and uracil, and backcrossed twice to confirm the linkage of *his^+^* and *cdc8*. The genomic sequence was confirmed. The strains created are: SH22 (*cdc8^D16A.K30A^*); SH41, SH104, SH105 (*cdc8^E104A^*); SH39, SH40 (*cdc8^R121A^*); SH43, SH44 (*cdc8^D131A^*); SH34 (*cdc8^E138A^*).

#### Fluorescent protein strains

Mutant *cdc8* strains were crossed into strains expressing the following cytoskeletal proteins with fluorescent protein tags (supplementary material Table S1): α-actinin (*ain1-mEGFP*, JW1144), coronin (*crn1-GFP*, FC661), fimbrin (*fim1-mEGFP*, JW1142), formin (*cdc12-3xGFP*, KV344), myosin 1 (*myo1-mGFP*, MLY422), myosin 52 (*myo52-3xYFP*, MLY681), myosin regulatory light chain (*rlc1-mCherry*, MLY744), tubulin (*atb2-mRFP*, MLY1065), and LifeAct (*pAct1-LifeAct-GFP*, KV588). Wildtype and mutant *cdc8* strains were crossed and colonies following random spore analysis were selected using nutritional, fluorescent and or kanamycin resistance, as appropriate. Except for *myo1-mGFP* (MLY422), *crn1-GFP* (FC661) and *LifeAct-GFP* (KV587) we constructed gene replacement *cdc8^+^* strains expressing the fluorescent protein (*grcdc8^+^* in supplementary material Table S1). The *grcdc8^+^* strains are *ura4^+^*. We did not note a significant difference in the actin cytoskeleton between the *grccdc8^+^, ura^+^* strains and the parental *cdc8^+^ura^−^* strains.

### Microscopy

#### Staining

For microscopy cells were grown in YEA and fixed at mid-log phase. For staining with DAPI and Calcofluor cells were fixed and stored in 70% ethanol (Forsburg, 2003a; Mitchison and Nurse, 1985). To visualize the septum, ethanol-fixed cells were washed in PBS and resuspended in 5 µl 50-100 µg/ml Calcofluor (Sigma Life Science, St. Louis, MO, USA) in 50 mM sodium citrate, 100 mM sodium phosphate, pH 6.0) and incubated at ambient temperature for 5 min in the dark. For counterstaining with DAPI to visualize the nuclei, the cells were washed and resuspended in 2-5 µl PBS with the addition of 0.5 µl 50 µg/ml DAPI (Sigma Life Science). The samples were incubated for 5 min in the dark at ambient temperature. One µl of stained cells was mixed on a slide with 0.5 µl 1 mg/ml phenylenediamine in 50% glycerol, covered with a poly-l-lysine coated coverslip and sealed with clear nail polish.

Filamentous actin was visualized using Alexa Fluor 488 phalloidin or Rhodamine phalloidin (Life Technologies, Grand Island, NY, USA). Mid-log phase cells were fixed in 3.7% fresh paraformaldehyde (Electron Microscopy Sciences, Hatfield, PA, USA) for 5 min. at the growth temperature, washed three times in PBS, and stored in PBS with 0.01% NaN_3_ at 4°C for 1 week or less. To stain with phalloidin, 2 µl of fixed cells were permeabilized by vortexing in 100 µl 1% Triton X-100 in PBS for 1 min, and washed 3× with PBS. Phalloidin was added to the permeabilized cells (4 µl of 0.2 U/µl phallodin) and incubated with gentle agitation for 50 min at room temperature. Counterstaining with DAPI and preparation of the slides was as described above.

The details of antibody preparation and purification, and indirect immunofluorescence are in the legend to supplementary material Fig. S2.

#### Fluorescence microscopy

Epifluorescence images were captured on a Nikon Optiphot 2 microscope fitted for epifluorescence using an Ushio USH-1020H mercury lamp with a Model HB-10101AF power supply and a DS epifluorescence illuminator with Chroma FITC and DAPI filters for imaging Alexa Fluor 488 phalloidin and Calcofluor/DAPI, respectively. We used a Nikon E-Plan 100×/1.25 Ph4DL oil immersion objective lens and a CoolSNAP *fx* camera (Photometrix, Tuscon, AZ, USA). Exposure was controlled by a Uniblitz Model VMM-D1 shutter driver (Vincent Associates, Rochester, NY, USA). IP Lab 4.0.8 (Scanalytics, Inc., Fairfax, VA USA) was used to control the microscope and its external devices. Depending on the stain intensity in the examined field, the exposure times were between 100 and 400 ms. Images of the same field were taken at different focal planes to visualize the full thickness of the cells. Binning was set to 1×1. The images were adjusted for brightness and contrast and merged using Image J 1.43u (Rasband, 1997-2012). Scale bars for all images were obtained by using an AO micrometer with 2 mm divisions subdivided into units of 10 µm.

#### Confocal microscopy

Confocal images were obtained at ambient temperature on an inverted Olympus IX Z spinning disk confocal microscope using an Olympus UPlan Fl 100×, 1.3NA oil immersion objective and a Hamamatsu EM-CCD C9100-02 digital camera (Hamamatsu Photonics, Hamamatsu, Shizuoka Prefecture, Japan). The binning was 1×1, 1000 ms exposure, E-gain=45, Z-slice=0.3 µm, 0.092 µm/pixel. The filters were GFP/Alx488 for GFP and Cy3 for m-Cherry.

#### Vacuole experiments

Cells were grown to mid-log phase and resuspended to 3×10^7^ cells/ml. The lipophilic dye, FM4-64 (EMD Millipore, Billerica, MA, USA), was added to 50 µM from a stock of 10 mM. Cells were incubated rotating at 4°C for 30 min to allow the dye to coat the cells. The cells were then washed in YEA at 4°C and resuspended to 5×10^6^ cells/m as described in (Brazer et al., 2000). Cells were grown shaking at room temperature and visualized at times 0–270 min to allow the cells to take up the dye by endocytosis. The vacuole sizes at each time point were analyzed using Image J 1.43u (Rasband, 1997-2012) by measuring the diameter of each vacuole (using the conversion 1 pixel=0.0535 µm). The lengths were rounded to the nearest tenth of a µm and sorted into groups to create a distribution.

#### Image analysis

We quantified the nuclear number and cell length in Calcofluor/DAPI stained micrographs, and other parameters using Image J 1.43u (Rasband, 1997-2012). The length of the cells was measured in pixels and converted to µm using a conversion factor of 1 pixel=0.0535 µm obtained using the AO micrometer above. The data were binned in groups to the nearest µm. Measurements of fluorescence intensity were corrected for background fluorescence. The statistics show the mean and standard deviation. Probabilities were obtained using a two-tailed *t*-test.

### Expression, purification and analysis of recombinant Cdc8p

A plasmid encoding wildtype Cdc8p, pET3a-AS-*cdc8* (gift of M. Lord, University of Vermont) (Studier et al., 1990), was used to produce Ala-Ser-Cdc8p. Mutant *cdc8* variants were constructed in pET3a-AS-*cdc8* by Mutagenex. The initial Met is cleaved after expression in *E. coli* since the second residue is Ala.

Recombinant wildtype and mutant AS-Cdc8p were expressed in *E.coli* BL21(DE3) using the autoinduction method (Studier, 2005) and purified using ammonium sulfate fractionation and anion ion exchange chromatography on DE52 cellulose (Whatman, GE Healthcare Life Sciences, Piscataway, NJ, USA) following established protocols (Hammell and Hitchcock-DeGregori, 1996; Hitchcock-DeGregori and Heald, 1987). The method was modified to maximize ammonium sulfate precipitation of AS-Cdc8p (45-75% or 30-75%, depending on the form). The purity was evaluated on SDS-PAGE gels and the concentration was determined using the tyrosine difference method (Edelhoch, 1967). Actin was purified from chicken pectoral muscle acetone powder using established methods (Hitchcock-De Gregori et al., 1982).

#### Actin binding assays

Actin 5 µM was mixed with 0.1 µM to 6 µM AS-Cdc8p in 20 mM MOPS, pH 7.0, 150 mM NaCl and 2 mM MgCl_2_ and cosedimented at 20°C at 60,000 rpm in a TLA100 rotor, Beckman TL-100 ultracentrifuge, for 30 min. (Hammell and Hitchcock-DeGregori, 1996). The pellets and supernatants were analyzed on 15% SDS-PAGE gels, stained with Coomassie blue, scanned and analyzed using an Image Scanner III (GE Healthcare Life Sciences) with Labscan 6.0 and Image Quant TL 7.0 image analysis software. The observed AS-Cdc8p/actin ratio was normalized to 1 by dividing the AS-Cdc8p/actin ratio obtained from densitometry by the AS-Cdc8p/actin ratio observed at saturation. The free AS-Cdc8p in the supernatant was calculated from standard curves for wildtype AS-Cdc8p. The binding constant, K_app_ and Hill coefficient (αH) were determined by fitting the data to the Hill equation using SigmaPlot (Jandel Scientific, San Rafael, CA, USA):
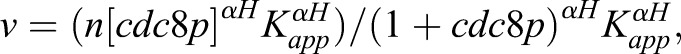
where α=fraction maximal AS-Cdc8p binding to actin, *n*=maximal AS-Cdc8p bound, and [*cdc8p*]=[AS-Cdc8p]_free_, and *αH*=Hill coefficient.

#### Circular dichroism measurements

Thermal stability was measured by following the ellipticity of AS-Cdc8p (0.2 mg/ml) at 222 nm in 0.5 M NaCl, 10 mM sodium phosphate pH 7.5, 1 mM EDTA at 0.2°C intervals from 0°C to 60°C using an Aviv model 400 CD-Spectrophotometer at the Robert Wood Johnson Medical School CD facility (Piscataway, NJ, USA). Ellipticity at 222 nm was normalized to a scale from 0 to 1. A value of 0.5 was defined as the observed melting temperature (T_M_) (Greenfield and Hitchcock-DeGregori, 1995).

## Supplementary Material

Supplementary Material
