# Supplementary material for: Evolutionarily conserved sites in yeast tropomyosin function in cell polarity, transport and contractile ring formation
Source: Biol Open. 2015 Jul 17;4(8):1040–51. doi: 10.1242/bio.012609 (PMC4542287; doi:10.1242/bio.012609)
Supplement: Supplementary Material [file supp_4_8_1040__index.html]

Evolutionarily conserved sites in yeast tropomyosin function in cell polarity, transport and contractile ring formation — Supplementary Material 

# Evolutionarily conserved sites in yeast tropomyosin function in cell polarity, transport and contractile ring formation

## BIO012609 Supplementary Material

- Supplementary Material
