# Supplementary material for: Evolutionarily conserved sites in yeast tropomyosin function in cell polarity, transport and contractile ring formation
Source: Biol Open. 2015 Jul 17;4(8):1040–51. doi: 10.1242/bio.012609 (PMC4542287; doi:10.1242/bio.012609)
Supplement: Supplementary Material [file supp_bio.012609_BIO012609supp.pdf]

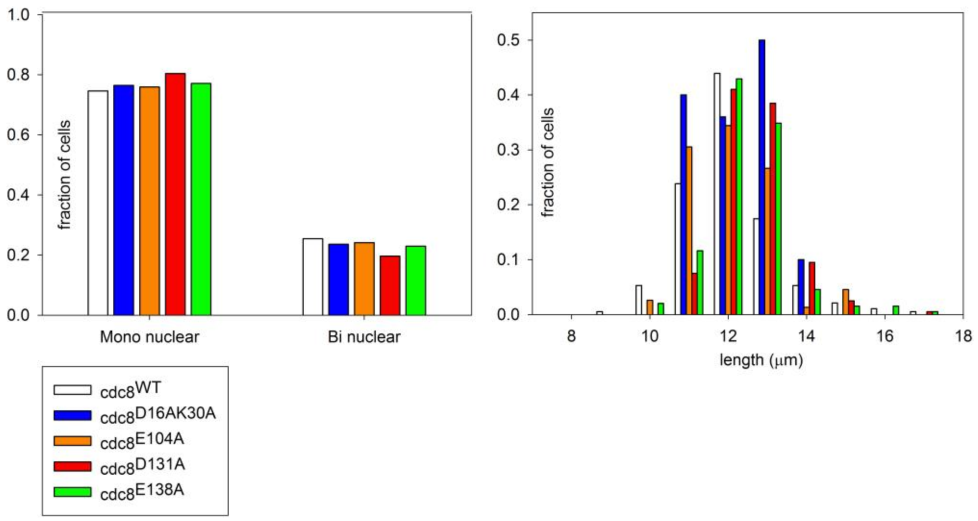

Fig. S1. Cell length and nuclear number of wildtype and *cdc8* mutants.

Wildtype and mutant strains were grown to mid-log phase in YEA at 30°C. Nuclear number and cell length were determined in Calcofluor/DAPI stained micrographs using Image J 1.43.

Left: Fraction of mononuclear and binuclear cells. The cell density was calculated using a hemocytometer.

Right: Length distribution of the cells. Cell length was measured in pixels and converted to μm using a conversion factor of 1 pixel = 0.0535 μm obtained using an AO micrometer (2 mm divisions subdivided into units of 10 μm). The distributions were plotted by rounding the lengths to nearest μm and sorting them into groups.

The results suggest that the *cdc8* mutations do not affect length or nuclear number.

Morphometrics of wildtype and mutant strains (mean and standard deviation)

Strain	Mononuclear	Binuclear	Length (mm)
cdc8^{WT}	0.75 (0.04)	0.25 (0.04)	12.0 (0.8)
cdc8^{D16AK30A}	0.76 (0.01)	0.24 (0.01)	12.9 (0.2)
cdc8^{E104A}	0.75 (0.04)	0.25 (0.04)	12.1 (0.2)
cdc8^{D131A}	0.80 (0.03)	0.20 (0.03)	12.6 (0.1)
cdc8^{E138A}	0.77 (0.01)	0.23 (0.01)	12.4 (0.3)

Strains: *cdc8^{WT}* (SH30), *cdc8^{D16AK30A}* (SH22), *cdc8^{E104A}* (SH41), *cdc8^{D131A}* (SH43), *cdc8^{E138A}* (SH34). *cdc8^{R121A}* (SH39) was too abnormal to be quantified.

Nuclear number was determined from two (three for *cdc8*^{WT}) independent measurements, each >200 cells.

Length measurements were obtained from two (three for *cdc8*^{WT}) independent measurements, each comprising >50 binuclear, septated cells.

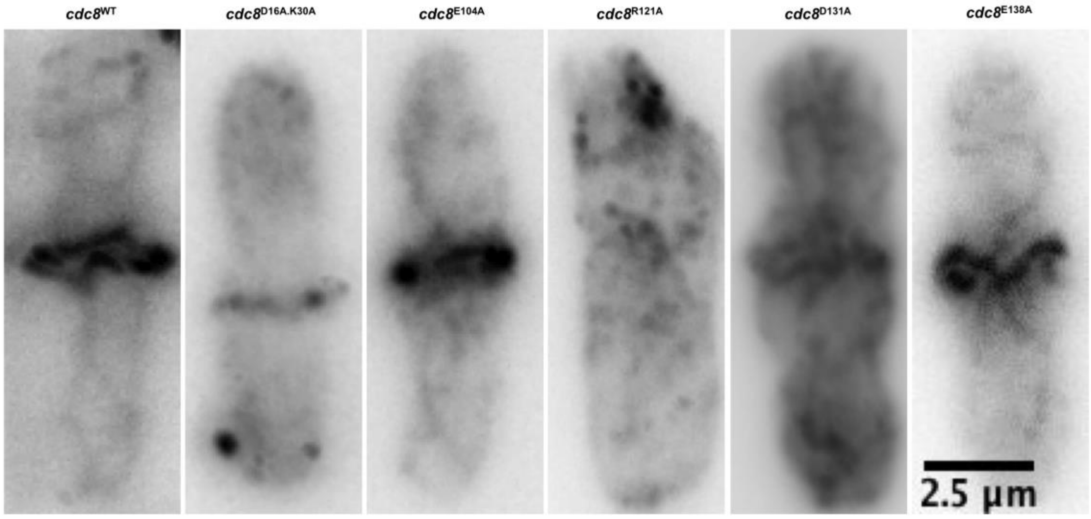

Fig. S2. Cdc8p visualized using indirect immunofluorescence.

The strains *cdc8*^{WT} (SH30), *cdc8*^{D16AK30A} (SH22), *cdc8*^{E104A} (SH41), *cdc8*^{R121A} (SH39), *cdc8*^{D131A} (SH43), and *cdc8*^{E138} (SH34) immunostained to show Cdc8p. Cdc8p was present in the contractile rings and cables of all strains, except *cdc8* ^{R121A}, which does not form these structures.

Antibody. Rabbit anti-Cdc8p was prepared by Covance (Princeton, NJ) and affinity purified by Genscript (Piscataway, NJ). Recombinant AlaSerCdc8p was expressed and purified from *E. coli* (Materials and Methods). Crude serum crossreacts with a single band in immunoblots of purified AS-Cdc8p or crude extracts.

Indirect immunofluorescence. For immunofluorescence imaging, cells were grown in YEA at 30°C overnight to mid-log phase. 0.6×10^7 cells were fixed in 4% paraformaldehyde (EM grade) at 30°C for 5 min, inverting once after 2.5 min. The cells from each strain were washed with PBS and resuspended in 140 µl 1.2 M sorbitol. 60 µl protoplasting solution was added (8.5 mg/ml Zymolase, 3 mg/ml final; 12 mg/ml lysing enzyme, final 5 mg/ml in 1.2M sorbitol). The cells were incubated at room temperature for 10-20 min and visually checked for protoplasting by mixing an aliquot 1:1 with 10% SDS. Protoplasted cells burst and disintegrate. To inactivate the protoplasting enzymes, 1 ml of 1% Triton was added and incubated for 2 min. Following centrifugation, cells were blocked by resuspension in 0.5 ml PBAL (10% BSA, 100 mM lysine HCl, 50 ng/ml Carbencillin, 1 mM NaN_3 in PBS) and incubated for 1 hr at room temperature, gently rocking. Following centrifugation, primary, affinity-purified anti-Cdc8p was added (100 µl, 1:10 in PBAL) and incubated overnight at 4°C. Cells were then spun down and washed thrice in 500 µl PBAL. Secondary antibody (Jackson ImmunoResearch Laboratories, West Grove, PA, 111-165-003, Cy3-goat anti-rabbit IgG (H+L)) was added (1:100 in PBAL) for 90 min. at room temperature. After six washes in PBAL the cells were ready for imaging. Cells were stored at 4°C and imaged within two days (details for fluorescence microscopy in Materials and Methods for fluorescence microscopy). The background with secondary antibody alone was negligible in the exposure times used (100 msec).

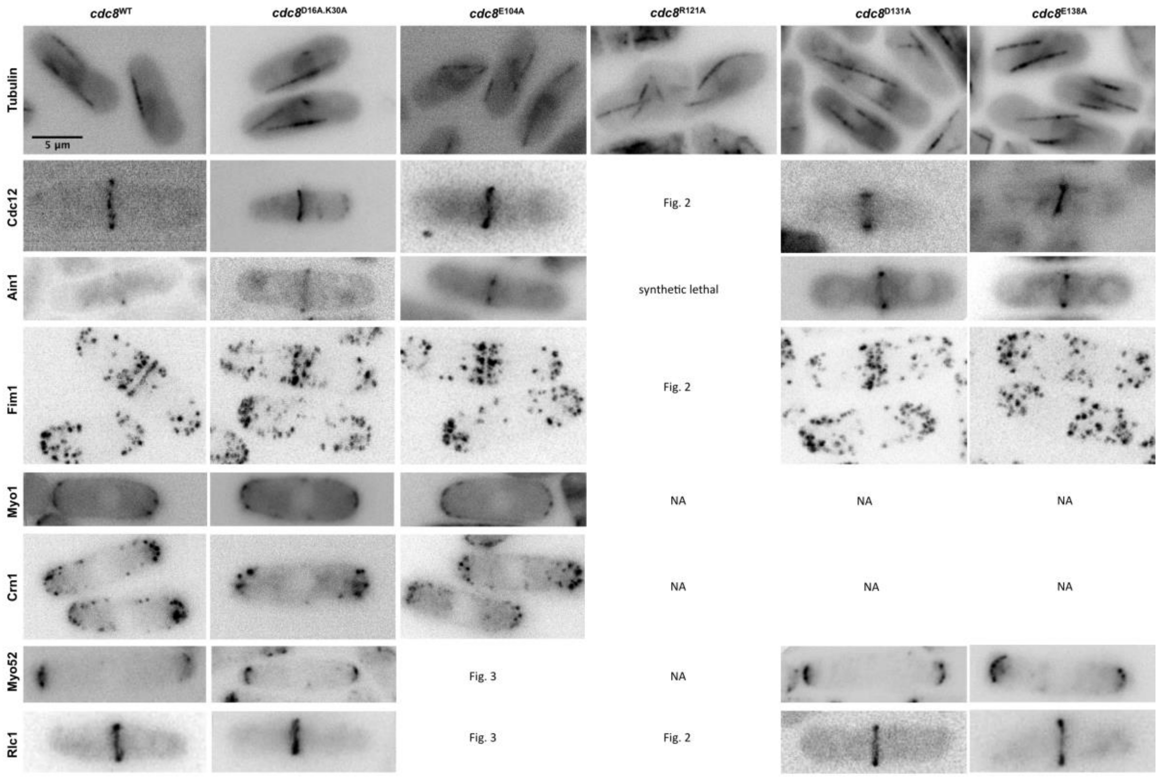

Fig. S3. Images of *cdc8* wildtype and mutant strains expressing fluorescent proteins.

All the images here show apparently normal phenotypes. Images that are shown in the main body of the paper are labeled with the figure number. NA = not available, the cross was not made. Strains used:
Row 1. Tubulin (mRFL-atb2): SH65, SH66, SH133, SH68, SH71, SH70

Row 2. Formin (cdc12-3xGFP): SH57, DH59, SH132, Fig. 2, SH62, SH64

Row 3. α-actinin (ain1-mEGFP): SH79, SH116, SH95, synthetic lethal, SH76, SH77-A

Row 4. Fimbrin (fim1-mEGFP): SH77, SH112, SH100, Fig. 2, SH74, SH75-A

Row 5. Myosin 1 (myo1-mGFP): MLY422, SH118, SH96, NA, NA, NA

Row 6. Coronin (crn1-GFP): FC661, SH129, SH128, NA, NA, NA

Row 7. Myosin 52 (myo52-3xYFP): SH107, SH121, Fig. 3, NA, SH108, SH109

Row 8. Myosin II regulatory light chain (rlc1-mCherry): SH49, SH50, Fig. 3, Fig. 2, SH54, SH56.

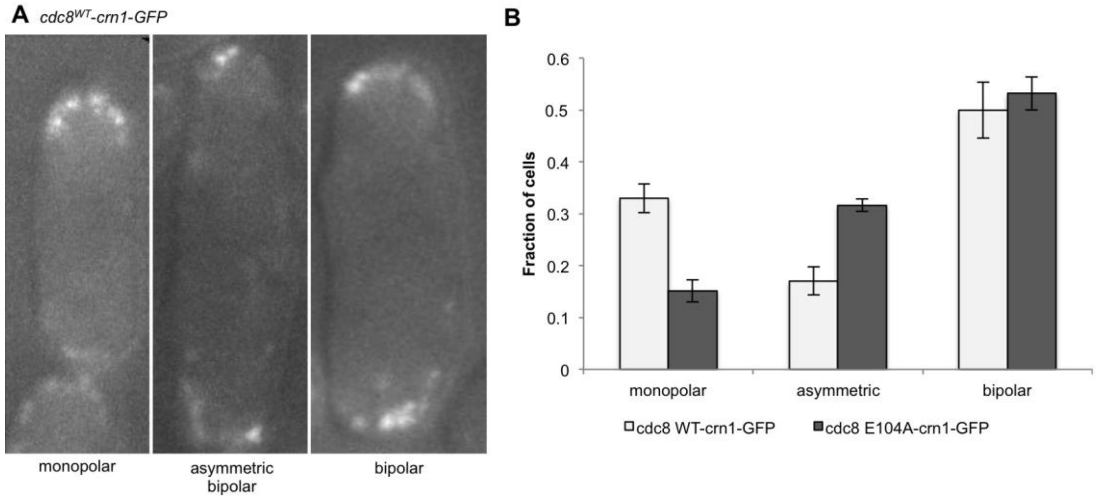

Fig. S4. Analysis of polar distribution of Crn1p-GFP patches in wildtype and E104A cells.

A. *cdc8^{WT}-crn1-GFP* cells (FC661) with monopolar, asymmetric, and bipolar patch distribution. The ratio of fluorescence intensity at the two ends of the cell was used to determine the polarity of the patch distribution: monopolar (≤0.33), bipolar (≥0.66), asymmetric bipolar (>0.33 but <0.66). See legend to Fig. 3. The cell lengths are: Wildtype: monopolar, 8.5±1.1 µm; asymmetric bipolar, 10.5±1.7 µm; bipolar, 10.8±1.8 µm. *cdc8^{E104A}*: monopolar, 8.4±0.5 µm; asymmetric bipolar, 9.2±1.7 µm; bipolar, 10.4±1.4 µm. Three trials, of n>70; n>210 across three experiments.

B. Fraction of cells with each patch distribution in *cdc8^{WT}* (FC661) and *cdc8^{E104A}-crn1-GFP* cells (SH128) based on fluorescence intensity quantification (n>78). There was a similar fraction of bipolar cells in *cdc8^{WT}* (0.50±0.05) and *cdc8^{E104A}* (0.53±0.03). *cdc8^{E104A}* had a decreased fraction of monopolar cells (0.15±0.02) and an increased fraction of asymmetric polar cells (0.31±0.01) compared to wildtype (monopolar, 0.33±0.03; asymmetric bipolar, 0.17±0.03).

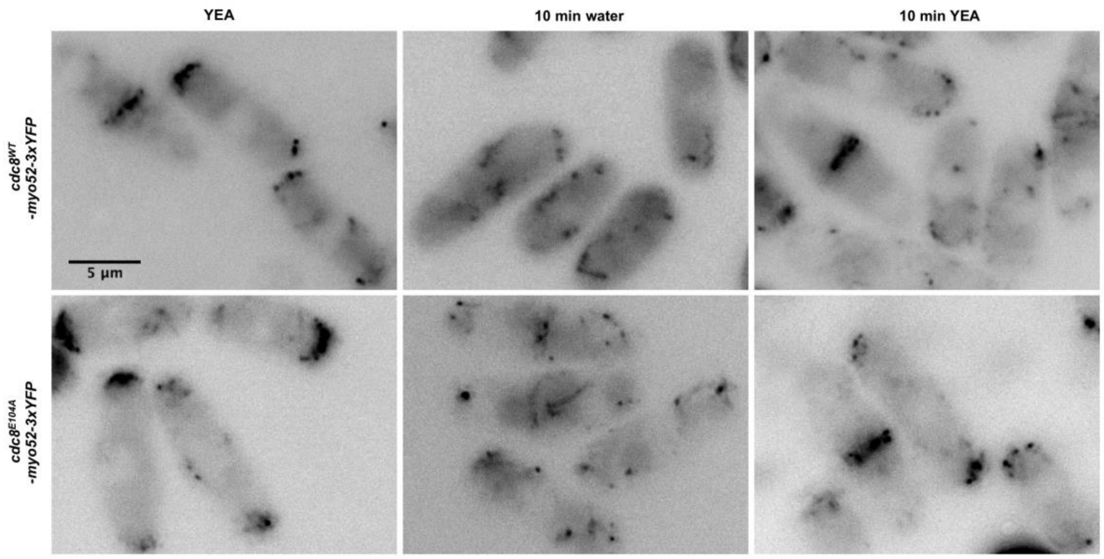

Figure S5. Myo52 depolarization and repolarization in response to osmotic stress.

cdc8^{WT} (SH107) and *cdc8*^{E104A} cells (SH98) expressing Myo52p-3x-YFP were grown in YEA. Myo52p is initially present at cell tips and in the contractile ring. Samples were spun down, washed, and resuspended in 1 ml H_2O and incubated at 30°C for 10 min. Myo52p-3x-YFP is visible in patches throughout the cell since it has redistributed to facilitate vacuole fusion. Samples were spun down, resuspended in YEA and incubated at 30°C for 10 min. Myo52p-3x-YFP returned to its normal distribution.

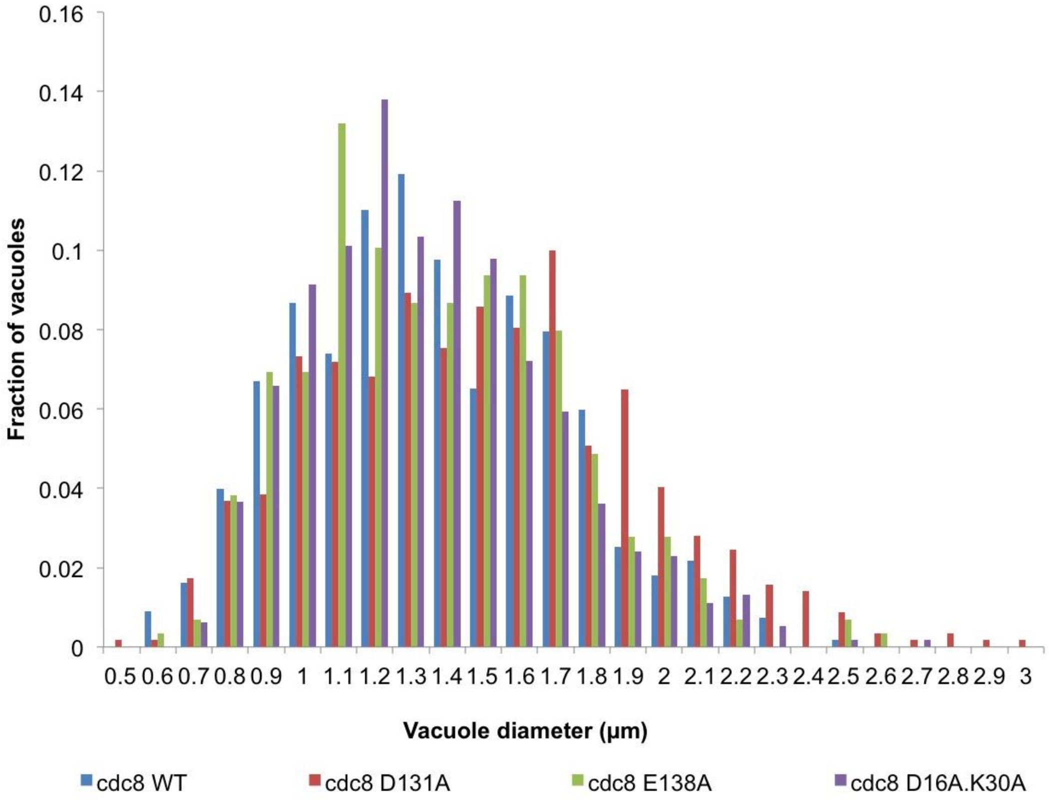

Figure S6. Size distribution of vacuoles in *cdc8*^{wt}, *cdc8*$^{D16A.K30A}$, *cdc8*^{D131A}, and *cdc8*^{D138A} cells.

Vacuole fusion is normal in *cdc8*$^{D16A.K30A}$ (SH22), *cdc8*^{D131A} (SH43) and *cdc8*^{D138A} cells (SH34).
The distribution of vacuole diameters was similar to *cdc8*^{WT} (SH30) (1.3±0.3 µm, n=275), *cdc8*$^{D16A.K30A}$ (1.4±0.3 µm, n=572), *cdc8*^{D131}(1.4±0.4 µm, n=288), and *cdc8*^{D138A} (1.4±0.4 µm, n=513).

Table S1. Strain List

Strain[1]		Genotype	Source/Reference
SH11	*h+*	*cdc8-27*:E129K *his5-D1 ura4-D18*	Cranz-Mileva et al. 2013
SH13	*h-*	*cdc8*$^{+}$*::his5*$^{\Delta c}$*::ura4*$^{+}$ *his5-D1 ura4-D18*	Cranz-Mileva et al. 2013
SH30[1]	*h+*	*grcdc8*$^{+}$ *::his5*$^{+}$*::ura4*$^{+}$ *his5-D1 ura4-D18*	Cranz-Mileva et al. 2013
SH22	*h+*	*cdc8*$^{D16A.K30A}$*::his5*$^{+}$*::ura4*$^{+}$ *his5-D1 ura4-D18*	Cranz-Mileva et al. 2013
SH41	*h-*	*cdc8*^{E104A}*::his5*$^{+}$*::ura4*$^{+}$ *his5-D1 ura4-D18*	This study
SH104,105	*h+*	*cdc8*^{E104A}*::his5*$^{+}$*::ura4*$^{+}$ *his5-D1 ura4-D18*	This study
SH39	*h-*	*cdc8*^{R121A}*::his5*$^{+}$*::ura4*$^{+}$ *his5-D1 ura4-D18*	This study
SH40	*h+*	*cdc8*^{R121A}*::his5*$^{+}$*::ura4*$^{+}$ *his5-D1 ura4-D18*	This study
SH43	*h+*	*cdc8*^{D131A}*::his5*$^{+}$*::ura4*$^{+}$ *his5-D1 ura4-D18*	This study
SH34	*h+*	*cdc8*^{E138A}*::his5*$^{+}$*::ura4*$^{+}$ *his5-D1 ura4-D18*	This study
MLY744	*h-*	*rlc1-mCherry:natR ade6-M216 leu1-32 his3-D1 ura4-D18*	M. Lord
SH129	*h+*	*rlc1-mCherry:natR ade6-M216 leu1-32 his3-D1 ura4-D18*	SH30xMLY744
SH49		*grcdc8*$^{+}$ *rlc1-mCherry:natR::his5*$^{+}$*::ura4*$^{+}$ *ade6-M216 leu1-32 his5-D1 ura4-D18*	SH30xMLY744
SH50		*cdc8*$^{D16A.K30A}$ *rlc1-mCherry:natR::his5*$^{+}$*::ura4*$^{+}$ *ade6-M216 leu1-32 his5-D1 ura4-D18*	SH22xMLY744
SH130		*cdc8*^{E104A} *rlc1-mCherry:natR::his5*$^{+}$*::ura4*$^{+}$ *ade6-M216 leu1-32 his5-D1 ura4-D18*	SH41xSH129
SH52		*cdc8*^{R121A} *rlc1-mCherry:natR::his5*$^{+}$*::ura4*$^{+}$ *ade6-M216 leu1-32 his5-D1 ura4-D18*	SH40xMLY744
SH54		*cdc8*^{D131A} *rlc1-mCherry:natR::his5*$^{+}$*::ura4*$^{+}$ *ade6-M216 leu1-32 his5-D1 ura4-D18*	SH43xMLY744
SH56		*cdc8*^{E138A} *rlc1-mCherry:natR::his5*$^{+}$*::ura4*$^{+}$ *ade6-M216 leu1-32 his5-D1 ura4-D18*	SH34xMLY744
KV344	*h-*	*cdc12-3xGFP::KanR leu1-32 his3-D1 ura4-D18 ade6-M216*	D. Kovar
SH131	*h+*	*cdc12-3xGFP::KanR leu1-32 his3-D1 ura4-D18 ade6-M216*	SH30xKV344
SH57		*grcdc8*$^{+}$ *cdc12-3xGFP::KanR::his5*$^{+}$*::ura4*$^{+}$ *leu1-32 his3-D1 ade6-M216 his5-D1 ura4-D18*	SH30xKV344
SH59		*cdc8*$^{D16A.K30A}$ *cdc12-3xGFP::KanR::his5*$^{+}$*::ura4*$^{+}$ *leu1-32 his3-D1 ade6-M216 his5-D1 ura4-D18*	SH22XKV344
SH132		*cdc8*^{E104A} *cdc12-3xGFP::KanR::his5*$^{+}$*::ura4*$^{+}$ *leu1-32 his3-D1 ade6-M216 his5-D1 ura4-D18*	SH41xSH131
SH60		*cdc8*^{R121A} *cdc12-3xGFP::KanR::his5*$^{+}$*::ura4*$^{+}$ *leu1-32 his3-D1 ade6-M216 his5-D1 ura4-D18*	SH40xKV344
SH62		*cdc8*^{D131A} *cdc12-3xGFP::KanR::his5*$^{+}$*::ura4*$^{+}$ *leu1-32 his3-D1 ade6-M216 his5-D1 ura4-D18*	SH44xKV344
SH64		*cdc8*^{E138A} *cdc12-3xGFP::KanR::his5*$^{+}$*::ura4*$^{+}$ *leu1-32 his3-D1 ade6-M216 his5-D1 ura4-D18*	SH34xKV344
MLY1064	*h-*	*atb2 promoter-mRFP-atb2 leu1-32 ura4-D18*	M. Lord
MLY1065	*h+*	*atb2 promoter-mRFP-atb2 ade6-M210 leu1-32 ura3-D18*	M. Lord
SH65		*grcdc8*$^{+}$ *atb2 promoter-mRFP-atb2::his5*$^{+}$*::ura4*$^{+}$ *leu1-32 his5-D1 ura4-D18*	SH30xMLY1064
SH66		*cdc8*$^{D16A.K30A}$ *atb2 promoter-mRFP-atb2::his5*$^{+}$*::ura4*$^{+}$ *leu1-32 his5-D1 ura4-D18*	SH22xMLY1064
SH133		*cdc8*^{E104A} *atb2 promoter-mRFP-atb2::his5*$^{+}$*::ura4*$^{+}$ *ade6-M210 leu1-32 ura3-D18 his5-D1 ura4-D18*	SH41xMLY1065
SH68		*cdc8*^{R121A} *atb2 promoter-mRFP-atb2::his5*$^{+}$*::ura4*$^{+}$ *leu1-32 his5-D1 ura4-D18*	SH40xMLY1064
SH71		*cdc8*^{D131A} *atb2 promoter-mRFP-atb2::his5*$^{+}$*::ura4*$^{+}$ *leu1-32 his5-D1 ura4-D18*	SH43xMLY1064
SH70		*cdc8*^{E138A} *atb2 promoter-mRFP-atb2::his5*$^{+}$*::ura4*$^{+}$ *leu1-32 his5-D1 ura4-D18*	SH34xMLY1064
JW1142	*h-*	*fim1-mEGFP-KanMX6 ade6-M210 leu1-32 ura4-D18*	J. Wu
SH77		*grcdc8*$^{+}$ *fim1-mEGFP-KanMX6::his5*$^{+}$*::ura4*$^{+}$ *leu1-32 his5-D1 ura4-D18*	SH30xJW1142

SH112		*cdc8*$^{D16A.K30A}$ *fim1-mEGFP-KanMX6::his5*$^{+}$*::ura4*$^{+}$ *leu1-32 his5-D1 ura4-D18*	SH22xJW1142
SH100		*cdc8*^{E104A} *fim1-mEGFP-KanMX6::his5*$^{+}$*::ura4*$^{+}$ *leu1-32 his5-D1 ura4-D18*	SH104xJW1142
SH75		*cdc8*^{R121A} *fim1-mEGFP-KanMX6::his5*$^{+}$*::ura4*$^{+}$ *leu1-32 his5-D1 ura4-D18*	SH40xJW1142
SH74		*cdc8*^{D131A} *fim1-mEGFP-KanMX6::his5*$^{+}$*::ura4*$^{+}$ *leu1-32 his5-D1 ura4-D18*	SH43xJW1142
SH75-A		*cdc8*^{E138A} *fim1-mEGFP-KanMX6::his5*$^{+}$*::ura4*$^{+}$ *leu1-32 his5-D1 ura4-D18*	SH34xJW1142
JW1144	*h-*	*ain1-mEGFP::KanMX6 ade6-M210 leu1-32 ura4-D18*	J. Wu
SH79		*grcdc8*$^{+}$ *ain1-mEGFP::KanMX6::his5*$^{+}$*::ura4*$^{+}$ *leu1-32 his5-D1 ura4-D18*	SH30xJW1144
SH116		*cdc8*$^{D16A.K30A}$ *ain1-mEGFP::KanMX6::his5*$^{+}$*::ura4*$^{+}$ *leu1-32 his5-D1 ura4-D18*	SH22xJW1144
SH95		*cdc8*^{E104A} *ain1-mEGFP::KanMX6::his5*$^{+}$*::ura4*$^{+}$ *leu1-32 his5-D1 ura4-D18*	SH104xJW1144
SH76		*cdc8*^{D131A} *ain1-mEGFP::KanMX6::his5*$^{+}$*::ura4*$^{+}$ *leu1-32 his5-D1 ura4-D18*	SH43xJW1144
SH77-A		*cdc8*^{E138A} *ain1-mEGFP::KanMX6::his5*$^{+}$*::ura4*$^{+}$ *leu1-32 his5-D1 ura4-D18*	SH34xJW1144
MLY422	*h-*	*myo1-mGFP::KanR leu1-32*	M. Lord
SH118		*cdc8*$^{D16A.K30A}$ *myo1-mGFP::KanR::his5*$^{+}$*::ura4*$^{+}$ *leu1-32 his5-D1 ura4-D18*	SH22xMLY422
SH96		*cdc8*^{E104A} *myo1-mGFP::KanR::his5*$^{+}$*::ura4*$^{+}$ *leu1-32 his5-D1 ura4-D18*	SH105xMLY422
MLY681	*h-*	*myo52-3xYFP::KanR ade6-M216 leu1-32 his3-D1 ura4-D18*	M. Lord
SH107		*grcdc8*$^{+}$ *myo52-3xYFP::KanR::his5*$^{+}$*::ura4*$^{+}$ *ade6-M216 leu1-32 his3-D1 his5-D1 ura4-D18*	SH30xMLY681
SH121		*cdc8*$^{D16A.K30A}$ *myo52-3xYFP::KanR::his5*$^{+}$*::ura4*$^{+}$ *ade6-M216 leu1-32 his3-D1 his5-D1 ura4-D18*	SH22xMLY681
SH98		*cdc8*^{E104A} *myo52-3xYFP::KanR::his5*$^{+}$*::ura4*$^{+}$ *ade6-M216 leu1-32 his3-D1 his5-D1 ura4-D18*	SH104xMLY681
SH108		*cdc8*^{D131A} *myo52-3xYFP::KanR::his5*$^{+}$*::ura4*$^{+}$ *ade6-M216 leu1-32 his3-D1 his5-D1 ura4-D18*	SH43xMLY681
SH109		*cdc8*^{E138A} *myo52-3xYFP::KanR::his5*$^{+}$*::ura4*$^{+}$ *ade6-M216 leu1-32 his3-D1 his5-D1 ura4-D18*	SH34xMLY681
FC661	*h+*	*crn1-GFP ura4*$^{+}$ *leu1-32 ade6-M216*	F. Chang
SH115	*h-*	*crn1-GFP ura4*$^{+}$ *leu1-32 ade6-M216*	MLY744xFC661
SH129		*cdc8*$^{D16A.K30A}$ *crn1-GFP::his5*$^{+}$*::ura4*$^{+}$ $^{+}$ *leu1-32 ade6-M216 his5-D1 ura4-D18*	SH22xSH115
SH128		*cdc8*^{E104A} *crn1-GFP::his5*$^{+}$*::ura4*$^{+}$ $^{+}$ *leu1-32 ade6-M216 his5-D1 ura4-D18*	SH41xFC661
KV587	*h-*	*pAct1 LifeAct-GFP::leu+ ade6-M216 leu1-32 ura4-D18*	
KV588	*h+*	*pAct1 LifeAct-GFP::leu+ ade6-M216 leu1-32 ura4-D18*	D. Kovar
SH122		*cdc8*$^{D16A.K30A}$ *pAct1 LifeAct-GFP::his5*$^{+}$*::ura4*$^{+}$ *ade6-M216 leu1-32 his5-D1 ura4-D18*	SH22xKV587
SH134		*cdc8*^{E104A} *pAct1 LifeAct-GFP::his5*$^{+}$*::ura4*$^{+}$ *ade6-M216 leu1-32 his5-D1 ura4-D18*	SH41xKV588
SH136		*cdc8*^{R121A} *pAct1 LifeAct-GFP::his5*$^{+}$*::ura4*$^{+}$ *ade6-M216 leu1-32 his5-D1 ura4-D18*	SH40xKV587
SH110		*cdc8*^{D131A} *pAct1 LifeAct-GFP::his5*$^{+}$*::ura4*$^{+}$ *ade6-M216 leu1-32 his5-D1 ura4-D18*	SH43xKV587
SH111		*cdc8*^{E138A} *pAct1 LifeAct-GFP::his5*$^{+}$*::ura4*$^{+}$ *ade6-M216 leu1-32 his5-D1 ura4-D18*	SH34xKV587
SH81		*cdc8-27*:E129K *rlc1-mCherry:natR ade6-M216 leu1-32 his3-D1 his5-D1 ura4-D18*	SH11xMLY744
SH83		*cdc8-27*:E129K *fim1-mEGFP::KanMX6 ade6-M210 leu1-32 his5-D1 ura4-D18*	SH11xJW1142

SH87		*cdc8-27*:E129K *cdc12-3xGFP::KanR leu1-32 his3-D1 ade6-M216his5-D1 ura4-D18*	SH11xKV344

[1]Strains designated as *grcdc8*$^+$ are strains used for marker reconstitution mutagenesis as described in methods. In most cases the fluorescent protein (FP) strains were crossed with SH30 as well as the mutant strains so that the all fluorescent protein strains are *ura4*$^+$*.* We did not note any morphological differences between the *grcdc8*$^+$ FP strains and the original FP strains obtained from colleagues. Consequently we did not create FP *grcdc8*$^+$ for *myo1, crn1, or LifeAct*.
